# Proteomics reveals ablation of PlGF increases antioxidant and neuroprotective proteins in the diabetic mouse retina

**DOI:** 10.1038/s41598-018-34955-x

**Published:** 2018-11-13

**Authors:** Madhu Sudhana Saddala, Anton Lennikov, Dennis J. Grab, Guei-Sheung Liu, Shibo Tang, Hu Huang

**Affiliations:** 1 Wilmer Eye Institute, Johns Hopkins University, Baltimore, Maryland United States of America; 2 Aier School of Ophthalmology, Central South University, Changsha, Hunan China; 3 Aier Eye Institute, Changsha, Hunan China; 4 School of Biomedicine, Far Eastern Federal University, Vladivostok, Russia; 5 The Department of Pathology, Uniformed Services University of the Health Sciences, Bethesda, MD United States of America; 6 The Department of Pathology, Johns Hopkins University, Baltimore, Maryland United States of America; 7 Menzies Institute for Medical Research, University of Tasmania, Hobart, Tasmania Australia

## Abstract

Placental growth factor (PlGF or PGF), a member of the vascular endothelial growth factor (VEGF) sub-family, plays a crucial role in pathological angiogenesis and inflammation. However, the underlying molecular mechanisms that PlGF mediates regarding the complications of non-proliferative diabetic retinopathy (DR) remain elusive. Using an LC-MS/MS-based label-free quantification proteomic approach we characterized the alterations in protein expression caused by PlGF ablation in the retinas obtained from C57BL6, Akita, PlGF^−/−^ and Akita.PlGF^−/−^ mice. After extraction and enzymatic digestion with Trypsin/LysC, the retinal proteins were analyzed by Q-Exactive hybrid Quadrupole-Orbitrap mass spectrometry. Differentially expressed proteins (DEPs) were identified in four comparisons based on Z-score normalization and reproducibility by Pearson’s correlation coefficient. The gene ontology (GO), functional pathways, and protein-protein network interaction analysis suggested that several proteins involved in insulin resistance pathways (Gnb1, Gnb2, Gnb4, Gnai2, Gnao1, Snap2, and Gngt1) were significantly down-regulated in PlGF ablated Akita diabetic mice (Akita.PlGF^−/−^ vs. Akita) but up-regulated in Akita vs. C57 and PlGF^−/−^ vs. C57 conditions. Two proteins involved in the antioxidant activity and neural protection pathways, Prdx6 and Map2 respectively, were up-regulated in the Akita.PlGF^−/−^ vs. Akita condition. Overall, we predict that down-regulation of proteins essential for insulin resistance, together with the up-regulation of antioxidant and neuroprotection proteins highlight and epitomize the potential mechanisms important for future anti-PlGF therapies in the treatment of DR.

## Introduction

Diabetic retinopathy (DR), a sight-threatening microvascular complication of diabetes myelitis (DM), remains the leading cause of vision loss world-wide in the adult population, especially in economically developed countries^1^. With the increasing number of people with DM, the prevalence of DR and diabetic macular edema (DME) is expected to grow^2^. Metabolic changes in the diabetic retina result in the altered expression pattern of several growth factors, neurotrophic factors, cytokines/chemokines, vasoactive agents, and inflammatory and adhesion molecules, resulting in vascular lesions and cell death^3–5^. Emerging evidence suggests that retinal neurodegeneration is an early event in the pathogenesis of DR, which could participate in the development of microvascular abnormalities^6,7^.

Placenta growth factor (PlGF) is a member of vascular endothelial growth factor (VEGF) family proteins that were first discovered in human placental cDNA in 1991^8^. Over two decades of scientific research and development have increased our understanding of the PlGF biological function. Despite the high level of expression in the placenta, the ablation of PlGF in mice did not compromise the healthy embryonic development or adverse postnatal health effects^9^. Delivery of recombinant PlGF homodimer, PlGF-VEGFA heterodimer significantly promoted angiogenesis in ischemic conditions through FLT1^10^. Furthermore, many other cell types express PlGF in pathological conditions, including retinal pigment epithelial cells (RPE)^11^. This up-regulation is due not only to hypoxia but to responses to other stimuli including nitric oxide^12^, cytokines, such as interleukin-1 (IL-1), TNF-α^13^ and transforming growth factor-β1 (TGF-β)^14^. The observation that endothelial cells over-express PlGF under pathological angiogenesis conditions has also been confirmed^15^.

We reported that deleting PlGF in a C57BL/6-Ins2Akita/J (Akita) mouse line containing a dominant mutation induces spontaneous rapid onset diabetes^16^. In the retina, ablation of PlGF in the diabetic mice resulted in a decreased expression of diabetes-activated hypoxia-inducible factor (HIF)1α. Changes in VEGF pathway including expression of HIF1α, VEGF, VEGFR1–3, and levels of phospho (p)-VEGFR1, p-VEGFR2, and p–endothelial nitric oxide synthase in the retinas of diabetic PlGF^−/−^ mice, were also found. These changes occur without any noticeable effect on glucose balance or expression of intercellular adhesion molecule-1, vascular cell adhesion molecule-1, CD11b, and CD18^17^.

With much still to be learned regarding the biological roles of PlGF in DR, the transition from animal to human studies is currently underway in the form of 2phase II clinical trials designed to assess the therapeutic use of two anti-PlGF recombinant monoclonal antibodies in human DR patients: i.e., NCT03071068 and NCT03499223 (ThromboGenics, 2018)^18,19^. While the use of blocking PlGF antibodies for proteomic scale studies in humans is possible they are not without challenges.

Here we introduce a quantitative proteomic analysis approach based on label-free mass spectrometry (LFMS) to elucidate the beneficial role and molecular mechanisms of PIGF in DR using PIGF knockout mice. The study is based on retinal protein extracts from three genetically modified mouse strains with diabetic and PIGF knockout backgrounds. The advantages of LFMS for protein identification and quantification allowed a gel-free method without the use of isotopic labeling^20^. Furthermore, as DR affects the expression of many commonly used “housekeeping” proteins such as ACTB and Tubulin, an added advantage of the LDMS is that the number of peptide sequences rather than any relative quantification identifies proteins^21,22^, To date we have identified 3176 total proteins of which 107 were significantly different between the experimental group combinations tested (p < 0.05).

## Results

### Animals and diabetic conditions

**Figure 1:**
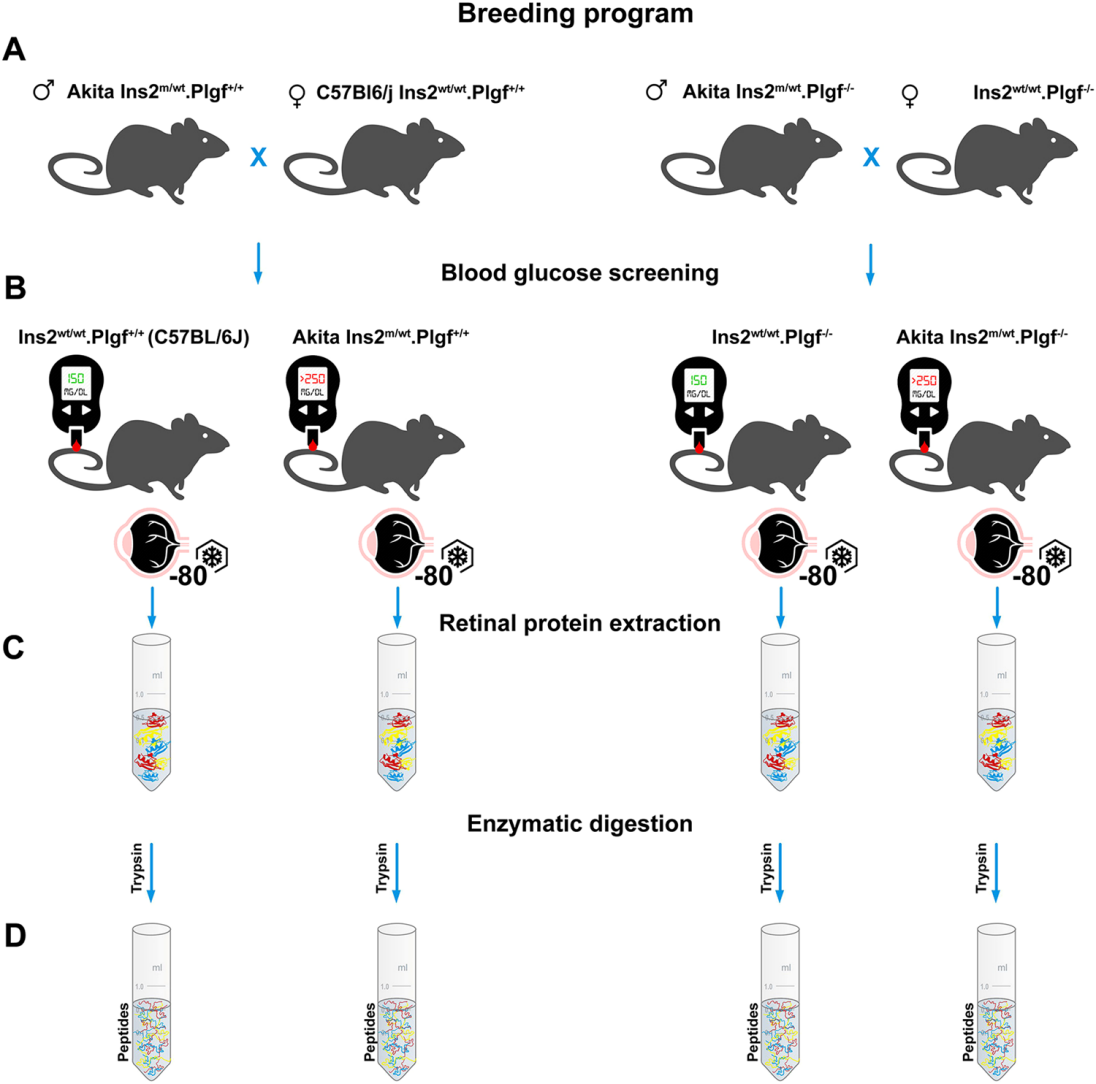
(**A**) Graphical representation of the animal breeding program; (**B**) screening of diabetic conditions; (**C**) retinal sample collection, (**D**) initial preparation of peptide digests. *Artwork design by Dmitry Rumyancev (*dmitry_r75@mail.ru*)*. *Copyright transferred to Macmillan Publishers Ltd*, *part of Springer Nature*, *under an open-access license Attribution 4*.*0 International (CC BY 4*.*0)*.

With four animals per study group, we monitored the body weight and blood glucose levels in control C57 (PlGF^+/+^) and Akita, Akita.PlGF^−/−^ and PlGF^−/−^ mice on a C57 background (Fig. 1). When compared to the C57 control animals of the same age, diabetic Akita mice demonstrated significant increases in blood glucose (BG) levels (p < 0.001) accompanied by decreases in body weight (p < 0.01). In contrast, no significant changes were found in blood glucose (p > 0.05) level or body weight (p > 0.05) in mice lacking PlGF; i.e., compare Akita.PlGF^−/−^ vs. Akita or PlGF^−/−^ vs. C57. Retinal protein fractions from all four conditions were also prepared LC/MS/MS analysis (below).

### Label-free proteomics data analysis

**Figure 2:**
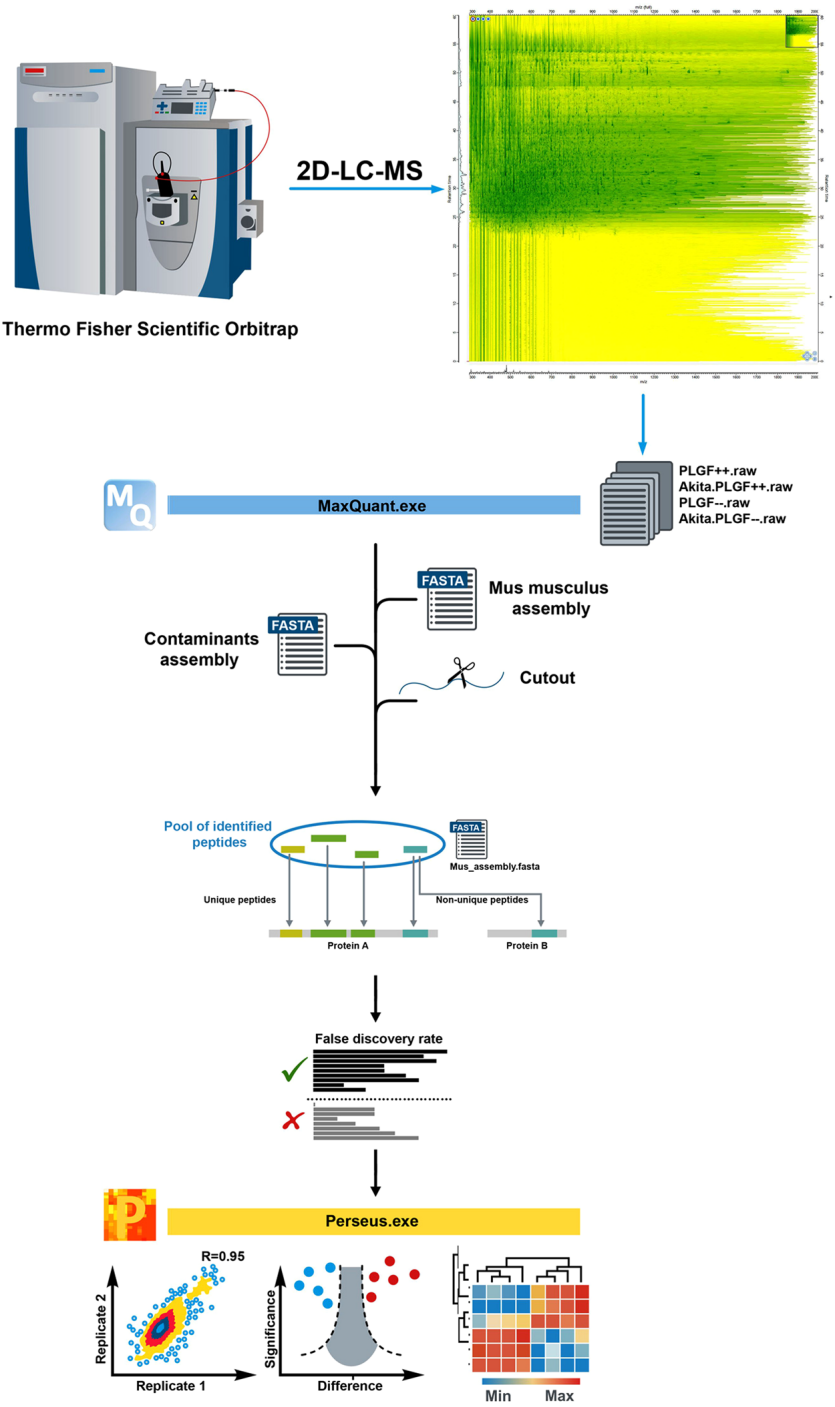
The overall workflow of our proteomics data analysis. The mass spectral (MS) raw data were analyzed with MaxQuant computational proteomics platform and its built-in Andromeda search engine. *Artwork design by Dmitry Rumyancev*.

The MaxQuant framework was used for proteomics data analysis, which is written in C# in the Microsoft.NET environment. Algorithmic sets of MaxQuant are freely accessible as source code, and the complete program (http://www.maxquant.org). We followed detailed instructions for installation and supporting programs by Cox *et al*.^23^. The four experimental data sets (Akita.PlGF^−/−^, PlGF^−/−^, Akita, and C57) were taken as a raw files for label-free quantification using MaxQuant version 1.6.01 (http://maxquant.org/). The overall proteomics data analysis workflow is represented in Fig. 2. From the dataset, MaxQuant calculated the number of quantified proteins, peptides, and sites. A total of 200102 msmsScans, 88071 msScans, 5990710 peaks, 465 oxidation(M)Sites, 668368 peptides and 794 proteins were identified (FDR < 0.01) along with mass, m/z, scans, and peaks, etc., for all data sets in the combined/text folder of the MaxQuant output directory.

### Perseus analysis pipeline

Perseus is software for shotgun proteomics data analysis, which helps to extract biologically meaningful information from processed raw files. It performs Bioinformatics analyses of the output of MaxQuant, and thus completes the proteomics analysis pipeline, allowing easy integration of an unlimited number of independent statistical tools, which can thus be combined in an analysis^24^. The experimental sample datasets (n = 4) were considered for statistical analysis: Akita.PlGF^−/−^ vs. Akita, Akita vs. C57, PlGF^−/−^ vs. C57 and Akita.PlGF^−/−^ vs. PlGF^−/−^. Because of Akita.PlGF^−/−^ was derived from PlGF^−/−^ background, whereas Akita from C57BL/6J, Akita. PlGF^−/−^ was not compared to C57, nor Akita to PlGF^−/−^ because the findings may be affected by the animal genetic background difference.

We determined that the LFQ (Label-Free Quantification) intensity of all combinations and intensity average be the same for all the samples and without bias. The correlation coefficient of LFQ intensities of the biological replicates was higher than 0.839. For the Akita.PlGF^−/−^ vs. Akita combination there was 0.839 low LFQ intensities between Akita.PlGF^−/−^ replicate 1 and Akita replicate 3, 0.937 high LFQ intensities between Akita.PlGF^−/−^ replicates 1 and 2. In Akita vs., C57 combination, 0.867 low LFQ intensities between Akita replicate four and C57 replicate 2, 0.943 high LFQ intensities between C57 replicates 3 and 4. In PlGF^−/−^ vs. C57 combination 0.876 low LFQ intensities between PlGF^−/−^ replicate 1 and C57 replicate 3, 0.945 high LFQ intensities between PlGF^−/−^ replicates 2 and 4. In Akita.PlGF^−/−^ vs. PlGF^−/−^ combination 0.877 low LFQ intensities between Akita.PlGF^−/−^ replicate 1 and 3, 0.948 high LFQ intensities between PlGF^−/−^ replicates 2 and 4, suggesting that the experiment has high repeatability and reliability (Suppl. Figs 1D, 2D, 3D and 4D). The hierarchical clustering analysis showed various components (Suppl. Figs 1A, 2A, 3A and 4A).

Differentially expressed proteins (DEPs) are screened for all combinations mentioned above based on fold-change (FC) >2 and p-value < 0.05.

**Figure 3:**
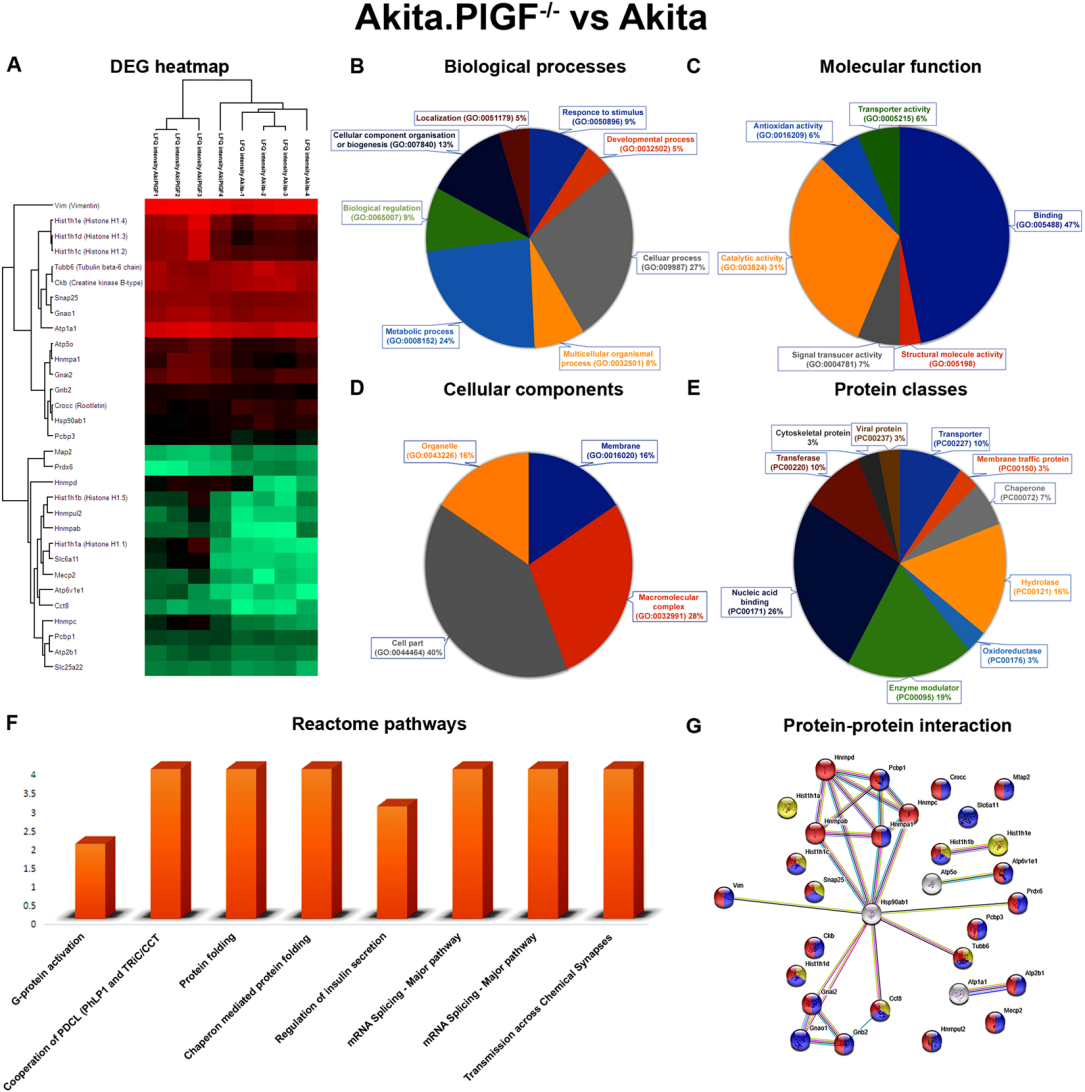
Functional annotation, Reactome pathways and protein-protein interaction network of Akita.PlGF^−/−^ vs. Akita. (**A**) Significantly up and down-regulated proteins were represented as a heat map. (**B**) The differentially expressed proteins (DEPs) are involved in the different biological process. (**C**) The differentially expressed proteins (DEPs) are involved in different molecular functions. (**D**) The DEPs are involved in various cellular components functions. (**E**) The DEPs are different classes of proteins. (**F**) The DEPs are involved in various Reactome biological pathways. (**G**) Highlighted are the various colors of DEPs involved in complex protein assembly (yellow) in a biological process, binding (blue) function of molecular function, membrane-bounded organelle (red) proteins in cellular component respectively.

**Figure 4:**
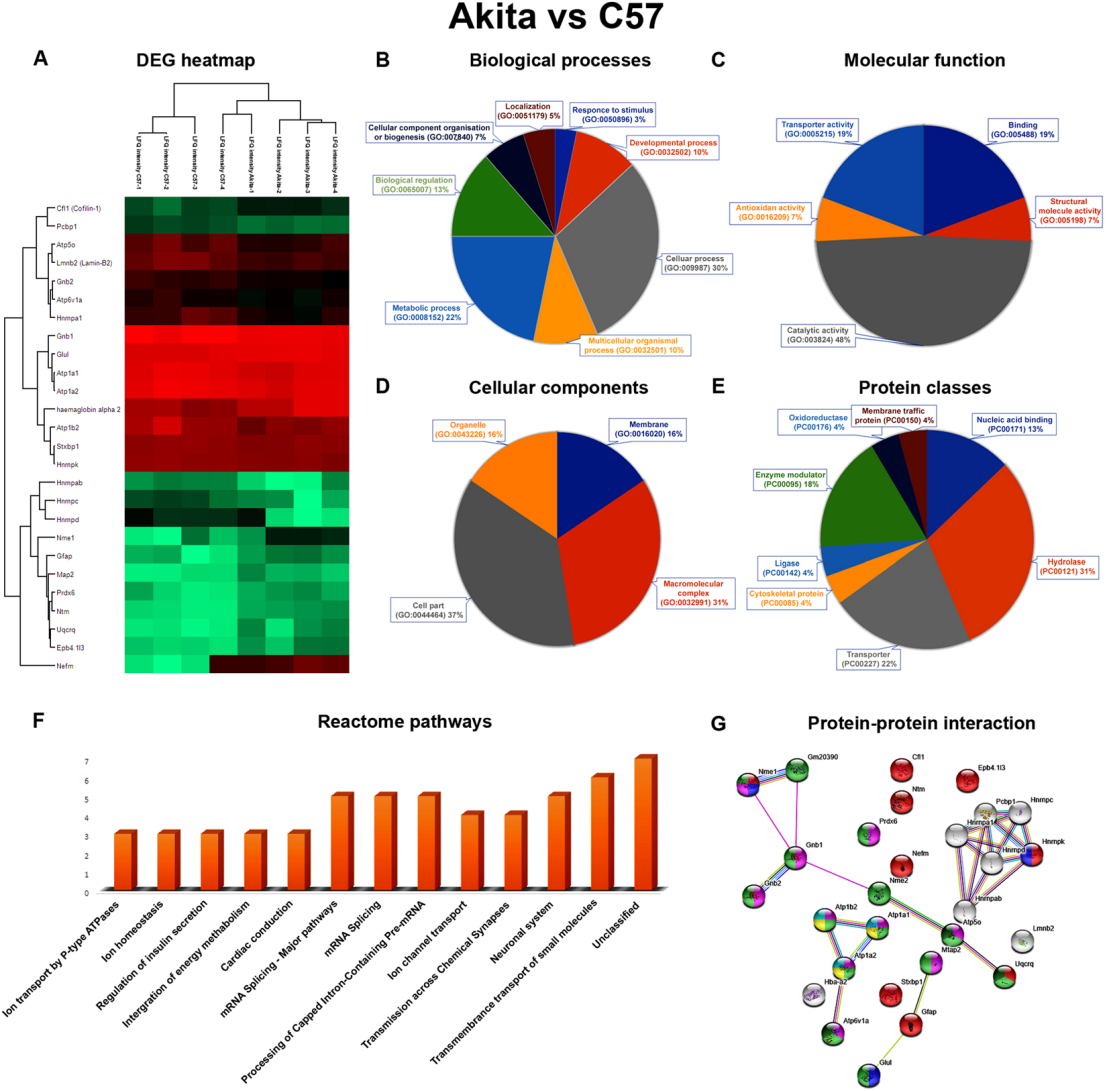
Functional annotation, Reactome pathways and protein-protein interaction network of Akita vs. C57. (**A**) Significantly up and down-regulated proteins were represented as a heat map. **(B**) The DEPs are involved in different biological processes. (**C**) The DEPs are involved in various molecular functions. (**D**) The DEPs are involved in various cellular components. (**F**) The DEPs are involved in various Reactome biological pathways. (**G**) Highlighted is the various colours of DEPs involved in nervous system development (red), response to glucose (blue) of biological process, catalytic activity (light green), hydrolase activity (pink) of molecular functions, insulin secretion (yellow), pancreatic secretion (cyan), oxidative phosphorylation (thick green) of KEGG pathways.

**Figure 5:**
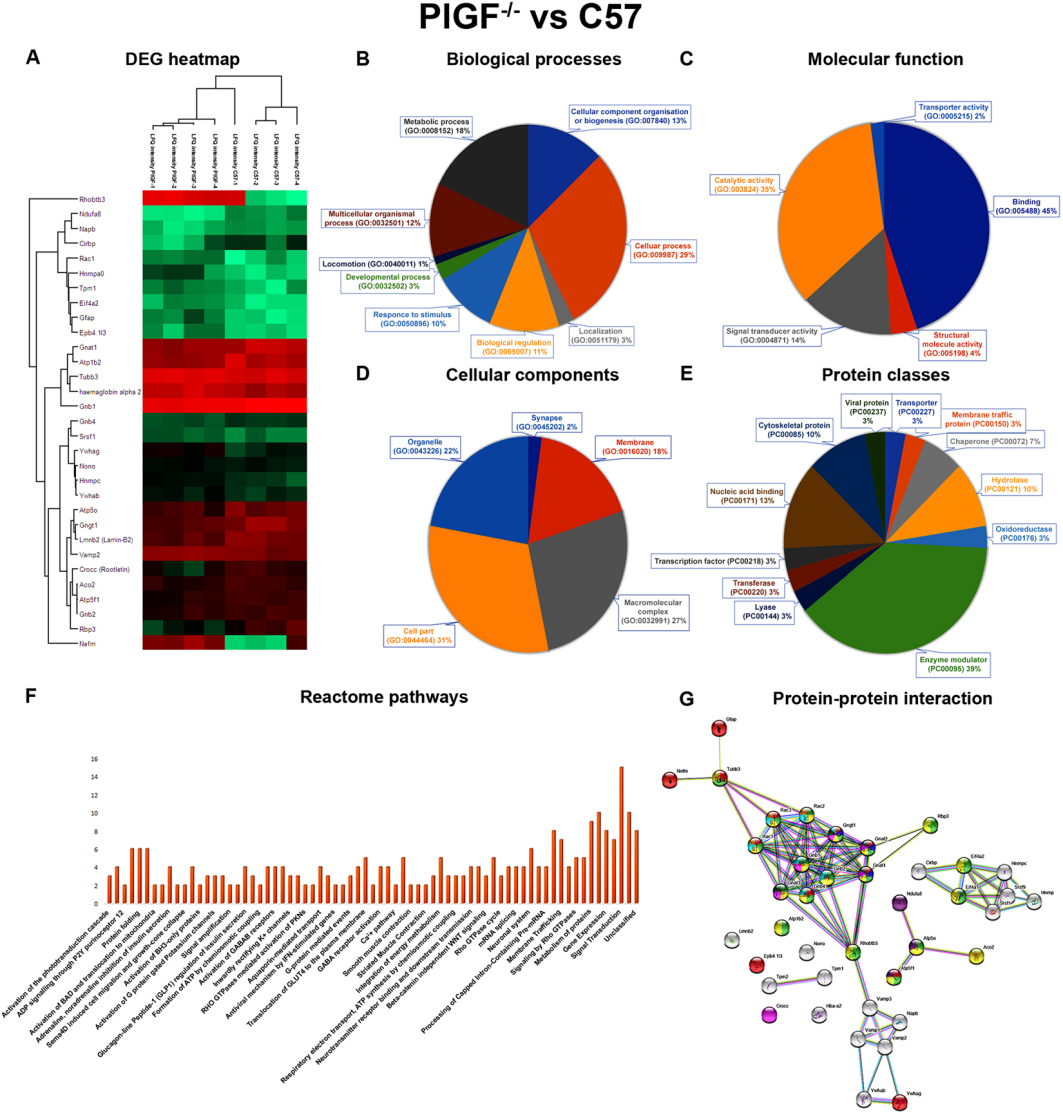
Functional annotation, Reactome pathways and protein-protein interaction network of PlGF^−/−^ vs. C57. (**A**) Significantly found up and down-regulated proteins were represented as a heat map. (**B**) The DEPs are involved in different biological processes. (**C**) The DEPs are involved in various molecular functions. (**D**) The DEPs are involved in various cellular components functions. (**E**) The DEPs are classified into various protein class. (**F**) The differentially expressed proteins are involved in various Reactome biological pathways. (**G**) Highlighted is the various colours of DEPs involved in nervous system development (red), eye photoreceptor cell differentiation (blue) of biological process, hydrolase activity (light green), catalytic activity (yellow) of molecular functions, photoreceptor inner segment (pink), photoreceptor outer segment (thick green) of cellular components, Ras signalling pathway (cyan), VEGF signalling pathway (orange), oxidative phosphorylation (magenta) of KEGG pathways.

**Figure 6:**
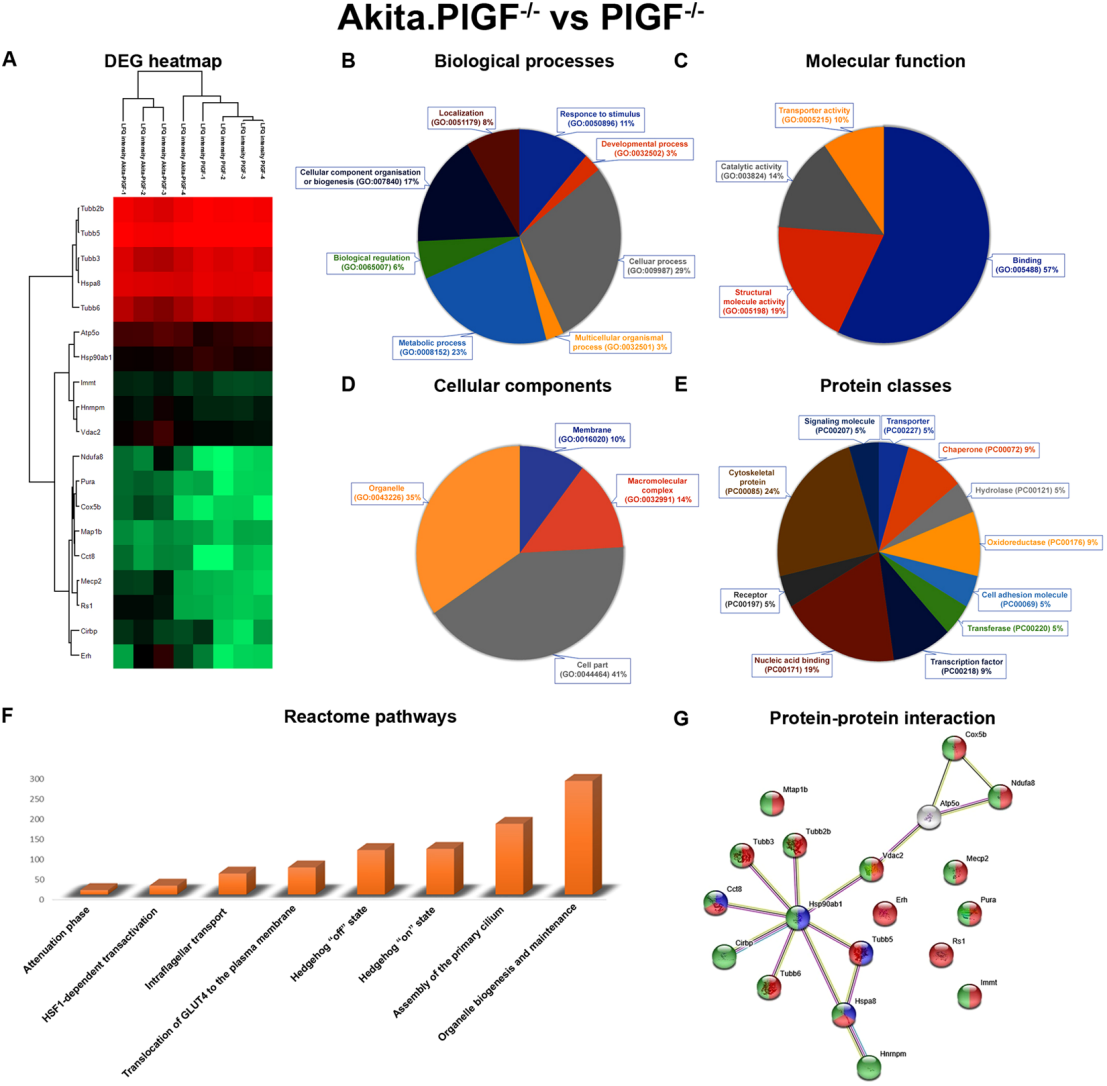
Functional annotation, Reactome pathways and protein-protein interaction network of Akita.PlGF^−/−^ vs PlGF^−/−^. (**A**) Significantly up and down-regulated proteins were represented as a heatmap. (**B**) The DEPs are involved in different biological processes. (**C**) The DEPs are involved in various molecular functions. (**D**) The DEPs are involved in various cellular components. (**F**) The DEPs are involved in various Reactome biological pathways. (**G**) Highlighted the various colors of DEPs involved in protein folding (blue) of biological process, binding (red) of molecular function, intracellular organelle part (green) of cellular component, Gap junction (green) of KEGG pathways.

The Akita.PlGF^−/−^ vs. Akita, combination has 31 DEPs: 6 up-regulated proteins, and 25 down-regulated proteins (Suppl. Table 2). The Akita vs. C57 combination has 26 DEPs: 15 up-regulated proteins and 11 down-regulated proteins (Suppl. Table 3). The PlGF^−/−^ vs. C57 combination has 31 DEPs: 15 up-regulated proteins and 16 down-regulated proteins (Suppl. Table 4). The Akita.PlGF^−/−^ vs. PlGF^−/−^ combination has 19 DEPs: 6 up-regulated proteins and 13 down-regulated proteins (Suppl. Table 5). All the comparisons of up and down-regulated proteins were represented as a heat map (Figs 3A, 4A, 5A and 6A). The DEPs of all combinations are used for gene annotation (GO) and pathways analysis.

### Functional classification and pathway analysis

The DEPs of all combinations were uploaded to the DAVID annotation tool using the complete mouse proteome as background. The GO terms were predicted based on Expression Analysis Systematic Explorer (EASE) < 0.1 and threshold count (TC) ≥ 2. The molecular functions, biological processes, cellular components protein classes, and pathways were predicted in the significantly-enriched GO terms of up and down-regulated proteins. The gene ontology functions identified for the DEPs in the Akita.PlGF^−/−^ vs. PlGF^−/−^ combination included biological processes (BP), molecular functions (MF), cellular components (CC), and protein classes and Reactome pathways. For biological processes, 27% of the proteins were predicted to be involved in cellular processes (GO:0032502) with another 24% in the metabolic process (GO:0008152) (Fig. 3B). In molecular functions, 47% and 31% of proteins were predicted to have binding (GO:005488) and catalytic activity (GO:003824), respectively (Fig. 3C). In cellular components, 48% of proteins belonged to the cell part (GO:0044464) (Fig. 3D). Another 26% and 19% of proteins had to do with nucleic acid binding (PC00171) and enzyme modulation (PC00095), respectively (Fig. 3E). Proteins involved in various Reactome biological pathways, included G-protein activation pathway, regulation of insulin secretion pathway and others were also found (Fig. 3F).

For the DEPs of Akita vs. C57 combination, involvement in various molecular functions, biological processes, cellular components, protein classes, and Reactome pathways were predicted.

For the biological process 30% and 22% of proteins were linked to cellular (GO:0009987) and metabolic (GO:0008152) processes respectively (Fig. 4B). Regarding molecular functions, 48% of the proteins were catalytic (GO:0003824), 19% had binding (GO:0005488), 19% transporter activity (GO:0005215) and other activities (Fig. 4C). Also, 37% of proteins belonged to the cell part (GO:0044464), 31% proteins belong to the macromolecular complex (GO:0032991) and others in cellular components (Fig. 4D). 31%, 22% of proteins belong to hydrolase (PC00121), transporter (PC00227) and other classes (Fig. 4E). The proteins are involved in mostly unclassified pathways, regulation of insulin secretion, transmission across chemical synapses and neuronal system pathways respectively (Fig. 4F).

The DEPs of PlGF^−/−^ vs. C57 combination is involved in various molecular functions, biological processes, cellular components, protein classes, and biological pathways. 29%, 18% of proteins are involved in various biological processes, such as cellular process (GO:0009987), metabolic process (GO:0008152) and other functions (Fig. 5B). 45% and 34% of proteins are involved in binding (GO:0005488), catalytic activity (GO:0003824) and other functions of molecular functions (Fig. 5C). The proteins are mainly involved in the cellular component process such as cell part (GO:0044464), macromolecular complex (GO:0032991) and organelle (GO:0043226) with 31%, 27% and 22% respectively (Fig. 5D). Most proteins (39%) belong to nucleic acid binding (PC00171) protein class (Fig. 5E). Reactome pathways results show that the proteins involved in activation of the phototransduction cascade, signal transduction, G-protein activation, adrenaline, noradrenaline inhibit insulin secretion and regulation of insulin secretion pathways (Fig. 5F).

The DEPs of Akita.PlGF^−/−^ vs. PlGF^−/−^ combination is involved in the various biological processes such as cellular process (GO:0009987) (29%), metabolic process (GO:0008152) (23%) and cellular component organization or biogenesis (GO:0071840) (17%) respectively (Fig. 6B). 57%, 19% of proteins are involved in binding (GO:0005488) and structural molecule activity (GO:0005198) in molecular functions (Fig. 6C). The proteins are mainly involved in the cellular component process such as cell part (GO:0044464) and organelle (GO:0043226) with 41% and 35% respectively (Fig. 6D). In protein class, 24% of proteins belong to cytoskeletal proteins (PC00085), 19% of proteins belong to nucleic acid binding (PC00171) proteins and others (Fig. 6E). The Reactome pathway results indicate that DEPs are involved in organelle biogenesis and maintenance, translocation of GLUT4 to the plasma membrane, Hedgehog ‘off’ state and Hedgehog ‘on’ state (Fig. 6F).

### Protein-protein network analysis

The DEPs of Akita.PlGF^−/−^ vs. Akita have been uploaded to STRING network. The network results showed that DEPs have total 30 nodes, 26 edges, cluster coefficient 0.497, average node degree:1.73, PPI enrichment p-value:1.17e-05 and involved in complex protein assembly (yellow) in biological process (GO:0006461), binding (blue) function of molecular function (GO:0005488), membrane-bounded organelle (red) proteins in cellular component (GO:0043227) (Fig. 3G). The DEPs of Akita vs. C57 have total 28 nodes, 27 edges, cluster coefficient 0.539, average node degree:1.73, PPI enrichment p-value: 2.62e-05 and involve in nervous system development (GO:0007399) (red), response to glucose (GO:0009749) (blue) of biological process, catalytic activity (GO:0003824) (light green), hydrolase activity (GO:0016787) (pink) of molecular functions, Insulin secretion (ko04911) (yellow), Pancreatic secretion (ko04972) (cyan), Oxidative phosphorylation (ko00190) (thick green) of KEGG pathways (Fig. 4G). The DEPs of PlGF^−/−^ vs. C57 have total 40 nodes, 87 edges, cluster coefficient 0.692, average node degree:4.35, PPI enrichment p-value: 9.93e-10 and involve in nervous system development (GO:0007399) (red), eye photoreceptor cell differentiation (GO:0001754) (blue) of biological process, hydrolase activity (GO:0016787) (light green), catalytic activity (GO:0003824) (yellow) of molecular functions, photoreceptor inner segment (GO:0001917) (pink), photoreceptor outer segment (GO:0001750) (thick green) of cellular components, Ras signaling pathway (ko04014) (cyan), VEGF signaling pathway (ko04370) (orange), Oxidative phosphorylation (ko00190) (magenta) of KEGG pathways (Fig. 5G). The up and down-regulated proteins of Akita.PlGF^−/−^ vs. PlGF^−/−^ have total 19 nodes, 14 edges, cluster coefficient 0.511, average node degree:1.47, PPI enrichment p-value: 0.0013 and involve in protein folding (GO:0006457) (blue) of biological process, binding (GO:0005488) (red) of molecular function, intracellular organelle part (GO:0044446) (green) of cellular component, Gap junction (ko04540) (green) of KEGG pathway (Fig. 6G).

**Figure 7:**
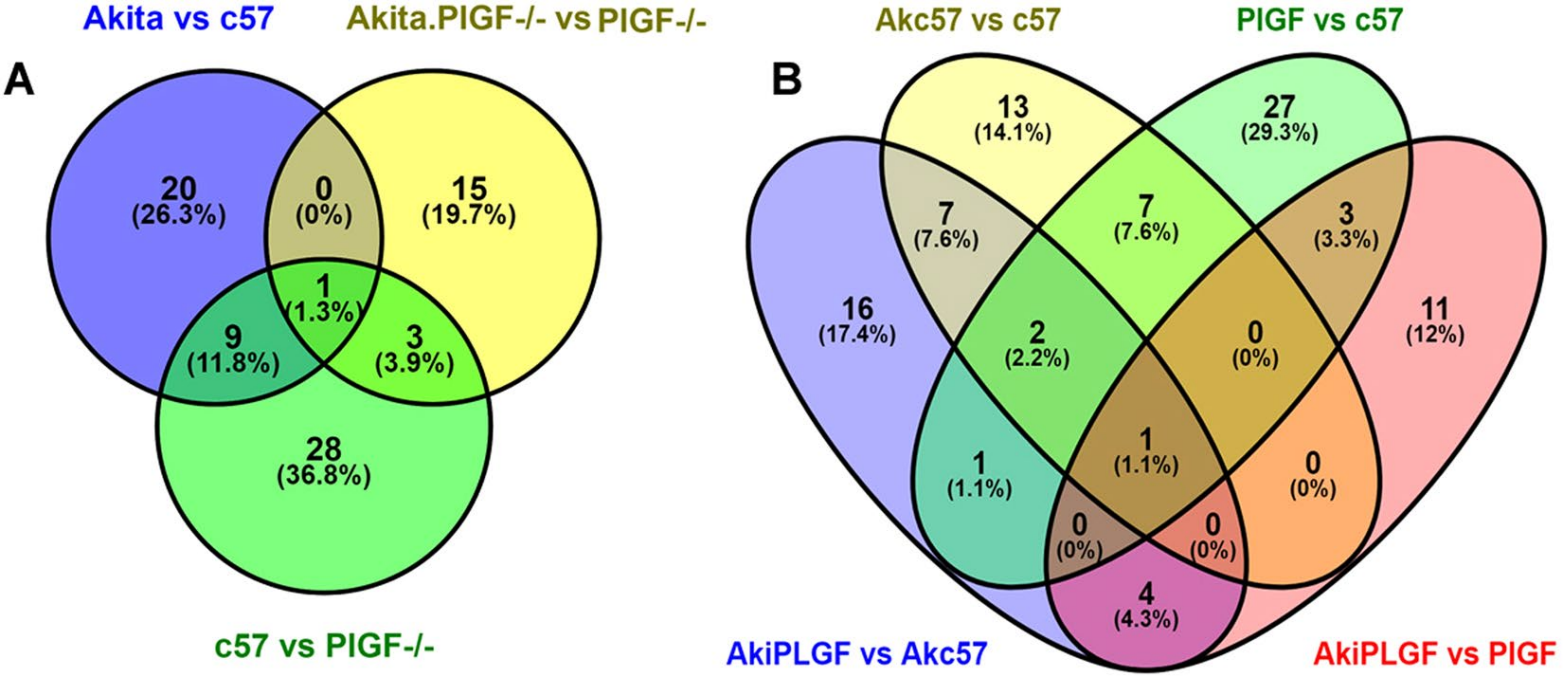
Comparisons of each group of the DEPs were illustrated by Venn diagrams. (**A**) The three groups of unique DEPs were represented as a Venn diagram. (**B**) The four groups of unique DEPs were represented as a Venn diagram.

The Venn diagrams showed that Akita vs. C57 had 30 DEPs, 20 (26.3%) proteins were unique, and 9 (11.8%) proteins were shared with PlGF^−/−^ vs. C57, only one (1.3%) protein was shared with other two groups. Akita. PlGF^−/−^ vs. Akita had 15 specific proteins, 3 proteins were shared with PlGF^−/−^ vs. C57, and one protein is shared with the other two groups (Fig. 7). We also provided the MS/MS spectrum and the confidently identified peptide components of Gnb1, Gnb2, Prdx6 and Map2 proteins (Suppl. Fig. 5).

## Discussion

In this study, we examined retinal proteome of the four mouse strains (Akita.PlGF^−/−^, Akita, C57, and PlGF^−/−^) using the label-free LC-MS/MS-based proteomics approach in order to understand better the molecular mechanisms that PlGF mediates in the complications of non-proliferative DR (NPDR). Using normalization by the Z-score method and correlation by the Pearson correlation coefficient we characterized the differentially expressed proteins (DEPs) between Akita.PlGF^−/−^ and Akita (31 proteins), Akita and C57 (26 proteins), PlGF^−/−^ and C57 (31 proteins), Akita.PlGF^−/−^ and PlGF^−/−^ (19 proteins). Given that the complete retinal protein extracts obtained from normal, diabetic and genetically manipulated mice were used for proteome analysis, it is possible these DEPs present any retinal cell types. Furthermore, given that ablation of PlGF in the transgenic mice provides the retinal protection against diabetic damages it is possible that these DEPs are involved in the protective retinal phenotypes as previously observed in Akita.PlGF^−/−^ mice. We highlight and discuss several relevant protein categories including insulin resistance pathway, antioxidant, cytoskeleton, chaperone, and neural protection.

The insulin resistance pathway proteins including Nucleotide Binding proteins (Gnb1, Gnb2, Gnb4, Gnai2, Gnao1), Snap25, Stxbp1, and Gngt1 are down-regulated due to PlGF ablation in Akita diabetics (Akita.PlGF^−/−^ vs. Akita) but up-regulated in Akita vs. C57. Gnb1, Gnb2, Gnb4, Gnai2, Gnao1 are classified as G-proteins that play a crucial role in the insulin signaling pathway. Incessant activation of G-proteins (polymorphism) results in insulin resistance and ultimately increases hepatic glucose output. Gnb gene dimorphism leads to increased cardiac potassium channel activity and α-adrenoceptor–mediated vasoconstriction thus resulting in the development and progression of hypertension, obesity, and insulin resistance in humans^25,26^. Gnb1, Gnb2, and Stxbp1 involved in regulation of insulin secretion pathway are up-regulated in Akita compared to the C57 group, the condition of which causes insulin resistance.

The role that down-regulation of synaptosomal-associated protein 25 (Snap25) found between the Akita.PlGF^−/−^ versus the Akita groups play in diabetes is suggested from literature. There is evidence that reduced expression of exocytotic genes (Stxbp1 and Snap25) leads to impaired insulin secretion^27^. On the other hand, the lowering of the trans-SNARE complex Snap25 involved in directly executing membranes fusion processes between the synaptic vesicle and plasma membranes appear to be associated with enhancement of insulin secretion^28^. These stimulatory and inhibitory influences may also involve the physical interaction of Snap25 with L-Type Ca^2+^ channels in β cells^29^. Our mass spectral results support the findings including the down-regulation of plasma membrane calcium-transporting ATPase1 (Atp2b1 protein)^29^.

Diabetes is often associated with increased oxidative stress and low energy levels that contribute to neuron microtubule meshwork degeneration, events that eventually leads to neuron apoptosis^30–32^. The up-regulation of several antioxidant and neural protection pathway proteins in the Akita.PlGF^−/−^ compared to Akita conditions is an intriguing and potentially important finding. The oxidoreductase class protein Peroxiredoxin-6 (Prdx6) is expressed in the majority of mammalian cells^33^. In the retina, solely Müller cells and astrocytes that play a crucial role in the maintenance of blood-retinal barrier function express Prdx6. Further, decreased Prdx6 has been reported in several disease conditions where the blood-retinal barrier is compromised. These include diabetic retinopathy (DR), exudative age-related macular degeneration (AMD) as well as arterial and venous occlusions^34^.

Tubb6 is the class of cytoskeletal protein that serves as a structural molecule and has the activities of binding, organization/biogenesis, and cellular process. Tubb6 allows cells to modify the organization of microtubules, regardless of the presence or absence of dystrophin, for the guidance and proper organization of microtubules^35^. Hsp90ab1 is the chaperone class protein that binds to other proteins, thereby stabilizing them in an ATP-dependent manner^36^.

Microtubule-associated proteins encoded by the Map2 gene are reported to comprise during microtubule assembly, a crucial step in the process of neurite formation. In rodents, Map2 are neuron-specific cytoskeletal proteins that determine and stabilize dendritic shape throughout neuron development^37^. Increased production of IL-1 beta (IL-1β), IL-6, or TNF-α decreases the Map2 expression and leads to neuronal cell death. Map2 upregulation increases neuronal cell survival and differentiation. Map2 also serves to stabilize microtubules (MT) growth by cross-linking microtubules with intermediate filaments as well as serve as a binding domain for the regulatory subunit II of cAMP-dependent protein kinase (PKA).

## Conclusion

Our results suggest that reduced insulin resistance in the Akita.PlGF^−/−^ mouse is potentially associated with the regulation of the Gnb family of proteins and P-type ATPases. This is important considering the role of these molecular players in regulating cell metabolism that allows retinal cells to better utilize glucose by reducing metabolic stress and ROS production. The data highlight significant roles for the neuron survival protein (Map2) and antioxidant defense protein (Prdx6) as well as roles for Tubb and Hsp protein that potentially increases retinal cell survival. Further functional studies are currently being done to identify other candidate proteins and the pathways involved to further explore the molecular mechanism of PlGF function and its target and off-target effects. Overall, how all the above molecular players intertwine in the end to accent the beneficial effects of targeting PIGF is a basis for future research.

## Materials and Methods

### The Mouse Strains

We used C57BL/6J [C57] mice (PlGF^+/+^ control] and Akita [C57BL/6-Ins2 < Akita >/J], Akita.PlGF^−/−^ **[**C57BL/6-Ins2 < Akita > /J.PlGF^−/−^**]** and PlGF^−/−^ **[**C57BL/6-PlGF^−/−^] mice were generated and maintained as previously described^38^. Briefly, Akita mice were crossed with PlGF^−/−^ mice^39^ in a C57BL/6J background for two generations to give birth to the progeny with the genotype of Akita.PlGF^−/−^ (Fig. 1A). The use of animals complied with the Association for Research in Vision and Ophthalmology (ARVO) statement for the use of animals in Ophthalmic and Vision Research and approved by the Institutional Animal Care and Use Committee of the Johns Hopkins University (Protocol number: M016M480). Mice were housed at the special pathogen-free (SPF) Cancer Research Building animal facilities at Johns Hopkins Hospital.

### Verification of diabetic conditions

Diabetic phenotypes were confirmed 4.5 weeks after birth by blood glucose >250 mg/dL (One-Touch Lifescan meter; Lifescan, Inc., Milpitas, CA) in a drop of blood from a tail puncture. Final blood glucose concentration and body weight were measured before sacrifice (Fig. 1B).

### Retinal samples collection

Mice were euthanized by intraperitoneal injection with ketamine hydrochloride (100 mg/kg body weight) and xylazine (4 mg/kg body weight). Mouse eyes were enucleated and the retinas carefully isolated free of the cornea, lens, vitreous humor and retinal pigment epithelium (RPE) on ice in cold phosphate-buffered saline (PBS, 0.1 M, pH = 7.2) under the dissection microscope (Stemi 2000-C, Zeiss). Retinas were snap-frozen with liquid nitrogen and then stored at −80 °C until further use.

### Protein extraction and peptide preparation

Retinas were homogenized in RIPA buffer (R0278-50ML; Sigma) supplemented with protease and phosphates inhibitor cocktail (Cell Signaling Technology). The retinal homogenates were centrifuged (12,000 g, 4 °C, 10 min) to remove tissue debris. The protein concentration of the supernatant was determined using Pierce BCA protein assay kit (Thermo Fisher Scientific, Cat#: 23225); 50-microgram of total protein from each sample was used for peptide preparation (Fig. 1C). DTT reduced proteins were alkylated by iodoacetamide (10 mM, in 50 mM Tris-HCl, pH8.0) at room temperature for 1 h. The protein samples were then digested with trypsin/LysC (Fig. 1D) (Promega, Cat#: V5073) in 25 mM ammonium bicarbonate solution at 37 °C for 12 h. The resultant peptides were washed and desalted with the desalting column (Pierce Spin-Tip) and eluted with 50 µl 5% acetonitrile +0.4% trifluoroacetic acid. The peptides mixtures were run through the ziptip C18 (Millipore) and then dried with SpeedVac concentrator.

### Mass spectrometry

The LC/MS/MS analyses of samples were carried out using a Thermo Scientific Q-Exactive hybrid Quadrupole-Orbitrap Mass Spectrometer and Thermo Dionex UltiMate 3000 RSLCnano System (Poolchon Scientific, Frederick, MD, USA). Peptide mixtures from each sample were loaded onto a peptide trap cartridge at a flow rate of 5 μL/min. The trapped peptides were eluted onto a reversed-phase PicoFrit column (New Objective, Woburn, MA) using a linear gradient of acetonitrile (3–36%) in 0.1% formic acid. The elution duration was 120 min at a flow rate of 0.3 μl/min. Eluted peptides from the PicoFrit column were ionized and sprayed into the mass spectrometer, using a Nanospray Flex Ion Source ES071 (Thermo) under the following settings: spray voltage, 1.6 kV, capillary temperature, 250 °C.

### Label-free proteomics data analysis

The mass spectral (MS) raw data were analyzed using the MaxQuant computational proteomics platform (version 1.6.1.0) and its built-in Andromeda search engine^40^. The LTQ-Orbitrap peptides were identified (main search peptide search = 4.5 ppm and 20 ppm for first search peptide tolerance, respectively) from the MS/MS spectra searched against the *Mus musculus* UniProtKB (83,598 entries) target database^41^ using the Andromeda search engine^42^.

This target database was also shared with the common contaminants and concatenated with the reversed versions of all sequences. In group-specific parameters, enzyme-specific was set to trypsin, and two missed cleavages were allowable; variable modifications were selected as fixed modification whereas carbamidomethylation (C) and oxidization (M) were set as fixed and variable modifications respectively were selected as variable modifications and 5 set to a maximum number of modifications per peptide. The type was set the standard; multiplicity was set to 1 to account for the label-free state, and label-free quantification was set LFQ, 2 set for minimum ration count^40^. The FDR was set to 0.01 for proteins, peptides and other parameters were set to default. Label minimum ratio count was set to 2, peptides for quantification was set to unique and razor and re-quantify to allow identification and quantification of proteins in groups for LFQ analysis.

First, MaxQuant were corrected for systematic inaccuracies of measured peptide masses and corresponding retention times of extracted peptides from the raw data. Then for peptide identification, mass and intensity of the peptide peaks in mass spectrometry (MS) spectrum were detected and assembled into three-dimensional (3D) peak hills over the m/z retention time plane, which was filtered by applying graph theory algorithms to identify isotope patterns. High mass accuracy was achieved by weighted averaging and through mass recalibration by subtracting the determined systematic mass error from the measured mass of each MS isotope pattern. Peptide and fragment masses (in case of an MS/MS spectrum) were searched in an organism-specific sequence database and then were scored by a probability-based approach termed peptide score. For limiting a certain number of peak matches by chance, a target-decoy-based false discovery rate (FDR) approach was utilized. The FDR was determined using statistical methods that account for multiple hypotheses testing. Also, the organism-specific database search includes not only the target sequences but also their reverse counterparts and contaminants, which help to determine a statistical cut-off for acceptable spectral matches. The assembly of peptide hits into protein hits to identify proteins is the next step, in which each identified peptide of a protein contributes to the overall identification accuracy. Also, an FDR-controlled algorithm called matching between runs was incorporated, which enables the MS/MS free identification of MS features in the complete data set for every single measurement, leading to an increase in the number of quantified proteins per sample^40^.

### Perseus analysis pipeline

The MaxQuant software generated output file “proteingroups.txt” was utilized for Pearson correlation, clustering and statistical analysis using Perseus software version 1.6.1.1^24^. Unverified hierarchical clustering of the LFQ values was carried out based on Euclidean distances on the Z-scored among mean values. For statistical analysis, two-samples t-test-based statistics with P < 0.05 was applied on Log2 transformed LFQ values and the minimum number of values “in at least one group” was three to assert proteins regulation as significant for the specific groups^43,44^.

### Functional classification and pathway analysis

Proteins determined to be differentially expressed based on the data in LFQ experiments were tabularize in Excel, and their gene names were used for functional annotation and pathways analysis. The DEPs were connecting to at least one annotation term each within the molecular function (MF), biological process (BP) and cellular component (CC) classes. In this study, DAVID Bioinformatics Resources 6.8 (https://david.ncifcrf.gov/)^45^ and GO Enrichment Analysis (http://geneontology.org/page/go-enrichment-analysis) were used for gene annotation of DEPs.

The protein list was uploaded into DAVID and searched for enrichment for GOBP term, and the results were filtered based on threshold count ≥2 and p-value <0.05.

### Protein-protein network analysis

The protein-protein network analysis is a wide-ranging approach to know the annotation of desire proteome^46^. The functional network analysis is helpful to discover a drug, to understand metabolic pathways and to predict or develop genotype-phenotype associations^47,48^. We have performed the protein-protein network analysis for all DEPs using STRING 10.5 database (https://string-db.org/). STRING tool classified the proteins according to the Gene Ontology (GO) categories such as biological process (BP), molecular function (MF), Cellular Component (CC) and KEGG (Kyoto Encyclopedia of Genes and Genomes database) pathways^49^. The tool Venny 2.1 (http://bioinfogp.cnb. csic.es/tools/venny/) was used to generate Venn diagrams.

### Statistical analysis

All numeric values were expressed as the mean ± standard deviation (SD) for the respective groups. Statistical analyses were performed using the MaxQuant software (http://www.maxquant.org). Student t-tests and Benjamini–Hochberg corrections (FDR) was used. A p-value of less than 0.05 was considered as a significant.

## Electronic supplementary material

Supplementary Tables 1–5; Supplementary Figures 1–5

## Data Availability

The dataset generated during this work have been deposited to the ProteomeXchange Consortium via PRIDE partner repository with the dataset identifier PXD010757.
