# Supplementary material for: Proteomics reveals ablation of PlGF increases antioxidant and neuroprotective proteins in the diabetic mouse retina
Source: Sci Rep. 2018 Nov 13;8:16728. doi: 10.1038/s41598-018-34955-x (PMC6233167; doi:10.1038/s41598-018-34955-x)
Supplement: Supplementary file 1 — Supplementary Tables 1–5; Supplementary Figures 1–5 [file 41598_2018_34955_MOESM1_ESM.pdf]

Proteomics reveals ablation of PlGF increases antioxidant and neuroprotective proteins in the diabetic mouse retina (Supplementary data)

Madhu Sudhana Saddala[1#], Anton Lennikov[1,4#] , Dennis J. Grab[5,6], Guei-Sheung Liu[7], Shibo Tang[2,3], Hu Huang[1,2,3]*

[1] Wilmer Eye Institute, Johns Hopkins University, Baltimore, Maryland, United States of America

[2] Aier School of Ophthalmology, Central South University, Changsha, Hunan, China

[3] Aier Eye Institute, Changsha, Hunan, China

[4] School of Biomedicine, Far Eastern Federal University, Vladivostok, Russia.

[5] The Department of Pathology, Uniformed Services University of the Health Sciences, Bethesda, MD, United States of America

[6] The Department of Pathology, Johns Hopkins University, Baltimore, Maryland, United States of America

[7] Menzies Institute for Medical Research, University of Tasmania, Hobart, Tasmania, Australia

*Corresponding author:

Hu Huang, PhD

Wilmer Eye Institute, Ophthalmology-Retinal Vascular Service

400 North Broadway St. Baltimore, MD, 21287

Hospital M017 Smith Building

Tel +1 (410)5020807

hhuang27@jhmi.edu

huang_hu@hotmail.com

# Madhu Sudhana Saddala and Anton Lennikov have contributed equally to this work.

Suppl. Table 1: Mouse blood glucose and body weight.

Mouse strain	Blood glucose (mg/dL)	Body weight (g)
C57	141	27
	163	26
	170	22
	157	23
Akita	628	22
	616	23
	439	26
	670	23
PlGF-/-	138	32
	161	30
	123	31
	151	32
Akita.PlGF-/-	504	24
	406	22
	539	19
	664	23

Suppl. Table 2: The list of significantly differentially expressed protein, gene name, MS/MS count, unique sequence coverage, molecular weight (MW), T-test difference, p-value, various KEGG name, and pathways in Akita.PlGF$^{-/-}$ group compared to Akita group (p-value<0.05).

Protein names	Gene names	Unique sequence coverage [%]	Mol. weight [kDa]	MS/MS count	Regulation	Student's T-test Difference AkiPLGF$^{-/-}$ _Akc57	Student's T-test Test statistic AkiPLGF$^{-/-}$ _Akc57	-Log Student's T-test p-value	KEGG	KEGG name
Peroxiredoxin-6	Prdx6	22.3	24.826	25	Up	1.82912302	5.092553817	2.6501	ko00360<br>ko00680<br>ko00940	Methane metabolism;<br>Phenylalanine metabolism;<br>Phenylpropanoid biosynthesis
Microtubule-associated protein; Microtubule-associated protein 2	Map2	9.7	49.264	46	Up	0.723612309	2.727109127	1.4644	ko04012<br>ko04722<br>ko04728<br>ko04310	ErbB signaling pathway<br>Neurotrophin signaling pathway<br>Dopaminergic synapse<br>Wnt signaling pathway
Tubulin beta-6 chain	Tubb6	4	50.09	174	Up	0.552398205	2.556084196	1.3651	ko04145<br>ko04540<br>ko05130	Gap junction<br>Pathogenic Escherichia coli infection<br>Phagosome
Rootletin	Crocc	18.3	226.94	143	Up	0.509349823	4.191491283	2.2411		
Heat shock protein HSP 90-beta	Hsp90ab1	8.3	83.28	83	Up	0.483288288	3.900802892	2.0981	ko04141<br>ko04612<br>ko04621<br>ko04626<br>ko04914<br>ko05200<br>ko05215	Antigen processing and presentation<br>NOD-like receptor signaling pathway<br>Pathways in cancer<br>Plant-pathogen interaction<br>Progesterone-mediated oocyte maturation<br>Prostate cancer<br>Protein processing in endoplasmic reticulum
Creatine kinase B-type	Ckb	62.7	42.713	311	Up	0.36639595	2.572327037	1.3746	ko00330	Arginine and proline metabolism
Guanine nucleotide-binding protein G(I)/G(S)/G(T) subunit beta-2	Gnb2	20.9	37.331	148	Down	-0.180434704	-2.728495349	1.4652	ko04062	Chemokine signaling pathway
Synaptosomal-associated protein 25	Snap25	71.4	23.315	276	Down	-0.303587437	-2.584567189	1.3818	ko04130	SNARE interactions in vesicular transport
Poly(rC)-binding protein 1	Pcbp1	12.1	37.497	68	Down	-0.339476585	-5.161235546	2.6793	ko03040	Spliceosome
Sodium/potassium-transporting ATPase subunit alpha-1	Atp1a1	25.8	112.98	395	Down	-0.356846809	-2.626044796	1.4059	ko04260<br>ko04960<br>ko04961<br>ko04964<br>ko04970<br>ko04971<br>ko04972<br>ko04973<br>ko04974	Aldosterone-regulated sodium reabsorption<br>Bile secretion<br>Carbohydrate digestion and absorption<br>Cardiac muscle contraction<br>Endocrine and other factor-regulated calcium reabsorption<br>Gastric acid secretion<br>Mineral absorption<br>Pancreatic secretion<br>Protein digestion and absorption

									ko04976<br>ko04978	Proximal tubule bicarbonate reclamation<br>Salivary secretion
Poly(rC)-binding protein 3	Pcbp3	20.8	24.117	82	Down	-0.35725832	-2.846988801	1.5331	ko00250<br>ko04270<br>ko04510<br>ko04810<br>ko04921	Alanine, aspartate and glutamate metabolism<br>Vascular smooth muscle contraction<br>Focal adhesion<br>Regulation of actin cytoskeleton<br>Oxytocin signaling pathway
Guanine nucleotide-binding protein G(o) subunit alpha	Gnao1	37.6	40.084	201	Down	-0.388885021	-2.47156149	1.3155	ko04730<br>ko04916<br>ko05142<br>ko05145	Chagas disease (American trypanosomiasis)<br>Long-term depression<br>Melanogenesis<br>Toxoplasmosis
Plasma membrane calcium-transporting ATPase 1	Atp2b1	4.7	134.75	48	Down	-0.422261715	-3.197607557	1.7291	ko04020<br>ko04970<br>ko04972	Calcium signaling pathway<br>Pancreatic secretion<br>Salivary secretion
ATP synthase subunit O, mitochondrial	Atp5o	37.1	23.363	79	Down	-0.423535824	-3.125266485	1.6893	ko00190<br>ko05010<br>ko05012<br>ko05016	Alzheimer's disease<br>Huntington's disease<br>Oxidative phosphorylation<br>Parkinson's disease
Guanine nucleotide-binding protein G(i) subunit alpha-2	Gnai2	22.5	40.489	103	Down	-0.461163998	-3.18320836	1.7212	ko04020<br>ko04062<br>ko04360<br>ko04530<br>ko04540<br>ko04670<br>ko04730<br>ko04740<br>ko04914<br>ko04916<br>ko04971<br>ko05142<br>ko05145<br>ko05146	Amoebiasis<br>Axon guidance<br>Calcium signaling pathway<br>Chagas disease (American trypanosomiasis)<br>Chemokine signaling pathway<br>Gap junction<br>Gastric acid secretion<br>Leukocyte transendothelial migration<br>Long-term depression<br>Melanogenesis<br>Olfactory transduction<br>Progesterone-mediated oocyte maturation<br>Tight junction<br>Toxoplasmosis
Vimentin	Vim	73.6	53.687	584	Down	-0.532655716	-2.526809502	1.3480	ko05014<br>ko05410<br>ko05412<br>ko05414	Amyotrophic lateral sclerosis (ALS)<br>Arrhythmogenic right ventricular cardiomyopathy (ARVC)<br>Dilated cardiomyopathy<br>Hypertrophic cardiomyopathy (HCM)
Mitochondrial glutamate carrier 1	Slc25a22	26.6	24.64	27	Down	-0.689178944	-3.51807772	1.9014		
Heterogeneous nuclear ribonucleoprotein A1	Hnrnpa1	30.3	38.833	65	Down	-0.78055954	-3.272488143	1.7700	ko03040	Spliceosome
Histone H1.2	Hist1h1c	21.2	21.266	115	Down	-1.250981331	-2.995659744	1.6172	ko05322<br>ko05034<br>ko04115k<br>o04914	Systemic lupus erythematosus<br>Alcoholism<br>p53 signaling pathway<br>Progesterone-mediated oocyte maturation
T-complex protein 1 subunit theta	Cct8	10.8	53.082	21	Down	-1.38865757	-3.498318032	1.8910		
Histone H1.4	Hist1h1e	16	21.977	119	Down	-1.393318176	-3.013641476	1.6272	ko05322<br>ko05034	Systemic lupus erythematosus<br>Alcoholism
Histone H1.3	Hist1h1d	9.5	22.099	105	Down	-1.504905224	-3.187679564	1.7237	ko05322<br>ko05034	Systemic lupus erythematosus<br>Alcoholism

Heterogeneous nuclear ribonucleoproteins C1/C2	Hnrnpc	28.8	34.384	51	Down	-1.816102505	-5.20234665	2.6967	ko03040	Spliceosome
V-type proton ATPase subunit E 1	Atp6v1e1	27.9	26.157	29	Down	-1.93034029	-2.750957456	1.4782	ko00190;<br>ko04145;<br>ko04966;<br>ko05110;<br>ko05120;<br>ko05323	Collecting duct acid secretion<br>Epithelial cell signaling in Helicobacter pylori infection<br>Oxidative phosphorylation<br>Phagosome<br>Rheumatoid arthritis<br>Vibrio cholerae infection
Heterogeneous nuclear ribonucleoprotein U-like protein 2	Hnrnpul2	16.1	84.939	40	Down	-1.97420311	-3.755246915	2.0245		
Methyl-CpG-binding protein 2	Mecp2	19	52.307	22	Down	-2.162194252	-3.561647541	1.9243	ko05016<br>ko04919<br>ko05202<br>ko04330<br>ko04110<br>ko04152	Huntington s disease<br>Thyroid hormone signaling pathway<br>Transcriptional misregulation in cancer<br>Notch signaling pathway<br>Cell cycle<br>AMPK signaling pathway
Histone H1.5	Hist1h1b	15.7	22.576	26	Down	-2.227953911	-3.3561123	1.8152	ko05322<br>ko04115	Systemic lupus erythematosus<br>p53 signaling pathway
Heterogeneous nuclear ribonucleoprotein A/B	Hnrnpab	29.6	33.816	30	Down	-2.522982121	-3.297190543	1.7834	ko03040	Spliceosome
Sodium- and chloride-dependent GABA transporter 3	Slc6a11	7.7	69.96	28	Down	-2.539692879	-3.292265714	1.7807		
Heterogeneous nuclear ribonucleoprotein D0	Hnrnpd	22.7	24.611	50	Down	-2.972328186	-3.282508326	1.7754	ko03040	Spliceosome
Histone H1.1	Hist1h1a	23	21.785	26	Down	-3.279434204	-4.172174855	2.2317	04115<br>04110<br>05322<br>05214<br>05200<br>04068	p53 signaling pathway<br>Cell cycle<br>Systemic lupus erythematosus<br>Glioma<br>Pathways in cancer<br>FoxO signaling pathway

Suppl. Table 3: The list of significantly differentially expressed protein, gene name, MS/MS count, unique sequence coverage, molecular weight (MW), T-test difference, p-value, various KEGG name, and pathways in Akita group compared to C57 group (p-value<0.05).

Protein names	Gene name	Unique sequence coverage [%]	Mol. weight [kDa]	MS/MS count	Regulation	Student's T-test Difference Akc57_ c57	Student's T-test Test statistic Akc57_ c57	-Log Student's T-test p-value	KEGG	KEGG name
Heterogeneous nuclear ribonucleoprotein D0	Hnrnpd	22.7	24.611	50	Up	2.259608746	2.549069207	1.7485	ko05014	Amyotrophic lateral sclerosis (ALS)
Heterogeneous nuclear ribonucleoproteins C1/C2	Hnrnpc	28.8	34.384	51	Up	1.582131863	2.60181487	1.6488	ko03040	Spliceosome
Heterogeneous nuclear ribonucleoprotein A/B	Hnrnpab	29.6	33.816	30	Up	1.428434372	2.629619451	1.4263		
Sodium/potassium-transporting ATPase subunit beta-2	Atp1b2	35.2	33.344	95	Up	0.736261368	2.537781636	1.6958	ko04260<br>ko04960<br>ko04961<br>ko04964<br>ko04970<br>ko04971<br>ko04972<br>ko04973<br>ko04974<br>ko04976<br>ko04978	Aldosterone-regulated sodium reabsorption<br>Bile secretion<br>Carbohydrate digestion and absorption<br>Cardiac muscle contraction<br>Endocrine and other factor-regulated calcium reabsorption<br>Gastric acid secretion<br>Mineral absorption<br>Pancreatic secretion<br>Protein digestion and absorption<br>Proximal tubule bicarbonate reclamation<br>Salivary secretion
Heterogeneous nuclear ribonucleoprotein A1 Heterogeneous nuclear ribonucleoprotein A1, N-terminally processed	Hnrnpa1	30.3	38.833	65	Up	0.686339378	3.301199097	3.4874	ko03040	Spliceosome
Lamin-B2	Lmnb2	31.7	67.317	126	Up	0.636933804	4.644004221	1.7731		
ATP synthase subunit O, mitochondrial	Atp5o	37.1	23.363	79	Up	0.630249023	3.515582565	1.9293	ko00190<br>ko05010<br>ko05012<br>ko05016	Alzheimer's disease<br>Huntington's disease<br>Oxidative phosphorylation<br>Parkinson's disease
Guanine nucleotide-binding protein G(I)/G(S)/G(T) subunit beta-1	Gnb1	45	37.377	334	Up	0.518396854	2.488870095	1.9654	ko04062;<br>ko04742;<br>ko04744	Chemokine signaling pathway<br>Photo transduction<br>Taste transduction
Poly(rC)-binding protein 1	Pcbp1	12.1	37.497	68	Up	0.486163139	6.353710521	1.5319	ko03040	Spliceosome
Guanine nucleotide-binding protein G(I)/G(S)/G(T) subunit beta-2	Gnb2	20.9	37.331	148	Up	0.440536022	5.2404351	2.1103	ko04062	Chemokine signaling pathway

V-type proton ATPase catalytic subunit A	Atp6v1a	31	68.325	160	Up	0.366929054	2.55926315	1.8044	ko00190<br>ko04145<br>ko04966<br>ko05110<br>ko05120<br>ko05323	Collecting duct acid secretion<br>Epithelial cell signaling in Helicobacter pylori infection<br>Oxidative phosphorylation<br>Phagosome<br>Rheumatoid arthritis<br>Vibrio cholerae infection
Sodium/potassium-transporting ATPase subunit alpha-2	Atp1a2	1.4	103.58	296	Up	0.365712643	2.748964859	1.3519	ko04260<br>ko04960<br>ko04961<br>ko04964<br>ko04970<br>ko04971<br>ko04972<br>ko04973<br>ko04974<br>ko04976<br>ko04978	Aldosterone-regulated sodium reabsorption<br>Bile secretion<br>Carbohydrate digestion and absorption<br>Cardiac muscle contraction<br>Endocrine and other factor-regulated calcium reabsorption<br>Gastric acid secretion<br>Mineral absorption<br>Pancreatic secretion<br>Protein digestion and absorption<br>Proximal tubule bicarbonate reclamation<br>Salivary secretion
Sodium/potassium-transporting ATPase subunit alpha-1	Atp1a1	25.8	112.98	395	Up	0.314900398	2.454267753	1.7570	ko04260<br>ko04960<br>ko04961<br>ko04964<br>ko04970<br>ko04971<br>ko04972<br>ko04973<br>ko04974<br>ko04976<br>ko04978	Aldosterone-regulated sodium reabsorption<br>Bile secretion<br>Carbohydrate digestion and absorption<br>Cardiac muscle contraction<br>Endocrine and other factor-regulated calcium reabsorption<br>Gastric acid secretion<br>Mineral absorption<br>Pancreatic secretion<br>Protein digestion and absorption<br>Proximal tubule bicarbonate reclamation<br>Salivary secretion
Heterogeneous nuclear ribonucleoprotein K	Hnrnpk	47.5	48.562	218	Up	0.207906246	3.248606535	1.3053	ko03040	Spliceosome
Syntaxin-binding protein 1	Stxbp1	34	67.568	257	Up	0.181161404	2.533583266	1.4770		
Glutamine synthetase	Glul	56	42.119	380	Down	-0.203300953	-3.336068814	1.3670	ko00250<br>ko00330<br>ko00910<br>ko02020	Alanine, aspartate and glutamate metabolism<br>Arginine and proline metabolism<br>Nitrogen metabolism<br>Two-component system
Cofilin-1<br>Cofilin-2	Cfl1	57.8	18.559	71	Down	-0.602310181	-3.925211198	2.7127	ko04360<br>ko04666<br>ko04810	Axon guidance;<br>Fc gamma R-mediated phagocytosis<br>Regulation of actin cytoskeleton
Peroxiredoxin-6	Prdx6	22.3	24.826	25	Down	-0.915928364	-2.844815673	3.1471	ko00360<br>ko00680<br>ko00940	Methane metabolism<br>Phenylalanine metabolism<br>Phenylpropanoid biosynthesis
Haemoglobin subunit alpha	haemoglobin alpha2 Hba	62	15.112	115	Down	-0.935765743	-3.640587253	1.3257	ko05143<br>ko05144	African trypanosomiasis<br>Malaria
Microtubule-associated protein<br>Microtubule-associated protein 2	Map2	9.7	49.264	46	Down	-0.977025986	-3.571238769	1.9001		

Band 4.1-like protein3	Epb4.1l3<br>Epb41l3	10.5	97.596	31	Down	-1.136313438	-3.278261317	2.4527	ko04530	Tight junction
Neurotrimin	Ntm	13.9	34.954	16	Down	-1.203859806	-7.346191294	1.7855		
Cytochrome b-c1 complex subunit 8	Uqcrq	18.3	9.7681	18	Down	-1.340533257	-3.13707011	1.3544	ko00190<br>ko04260<br>ko05010<br>ko05012<br>ko05016	Alzheimer's disease<br>Cardiac muscle contraction<br>Huntington's disease<br>Oxidative phosphorylation<br>Parkinson's disease
Glial fibrillary acidic protein	Gfap	24.4	49.899	36	Down	-1.42773056	-2.661065428	1.4080		
Nucleoside diphosphate kinase A and B	Nme1<br>Gm2039<br>0Nme2	29.9	14.092	33	Down	-2.119158745	-3.052309265	1.3918	ko00230<br>ko00240	Purine metabolism<br>Pyrimidine metabolism
Neurofilament medium polypeptide	Nefm	1.7	95.94	32	Down	-3.49536705	-3.232984617	1.3610	ko05014	Amyotrophic lateral sclerosis (ALS)

Suppl. Table 4: The list of significantly differentially expressed protein, gene name, MS/MS count, unique sequence coverage, molecular weight (MW), T-test difference, p-value, various KEGG name, and pathways in $PlGF^{-/-}$ group compared to C57 group (p-value<0.05).

**Protein names**	**Gene name**	**Unique sequence coverage [%]**	**Mol. weight [kDa]**	**MS/MS count**	**Regulation**	**Student's T-test Difference $PLGF^{-/-}$ _ c57**	**Student's T-test Test statistic $PLGF^{-/-}$ _ c57**	**-Log Student's T-test p-value**	**KEGG**	**KEGG name**
NADH dehydrogenase [ubiquinone] 1 alpha subcomplex subunit 8	Ndufa8	37.2	19.992	26	Up	1.762011528	8.051339072	3.7069	ko00190<br>ko05010<br>ko05012<br>ko05016	Alzheimer's disease<br>Huntington's disease<br>Oxidative phosphorylation<br>Parkinson's disease
Cold-inducible RNA-binding protein	Cirbp	44.2	18.607	34	Up	1.621991158	2.831672565	1.5244		
Retinol-binding protein 3	Rbp3	18.6	134.48	75	Up	1.191641808	4.157159713	2.2245		
Beta-soluble NSF attachment protein	Napb	26.2	33.557	39	Up	0.918866158	2.819272591	1.5173		
Rootletin	Crocc	18.3	226.94	143	Up	0.856175423	2.886512978	1.5556		
Guanine nucleotide-binding protein G(T) subunit gamma-T1	Gngt1	83.8	8.5278	63	Up	0.757047653	3.613410071	1.9513	ko04062<br>ko04744	Chemokine signaling pathway<br>Photo transduction
Guanine nucleotide-binding protein G(t) subunit alpha-1	Gnat1<br>Gnat2<br>Gnat3	46	39.966	257	Up	0.733029842	3.139031115	1.6969	ko04744	Photo transduction
Sodium/potassium-transporting ATPase subunit beta-2	Atp1b2	35.2	33.344	95	Up	0.659002304	2.53779854	1.3544	ko04260<br>ko04960<br>ko04961<br>ko04964<br>ko04970<br>ko04971<br>ko04972<br>ko04973<br>ko04974<br>ko04976<br>ko04978	Aldosterone-regulated sodium reabsorption<br>Bile secretion<br>Carbohydrate digestion and absorption<br>Cardiac muscle contraction<br>Endocrine and other factor-regulated calcium reabsorption<br>Gastric acid secretion<br>Mineral absorption<br>Pancreatic secretion<br>Protein digestion and absorption<br>Proximal tubule bicarbonate reclamation<br>Salivary secretion
Guanine nucleotide-binding protein G(I)/G(S)/G(T) subunit beta-1	Gnb1	45	37.377	334	Up	0.637954712	2.876404603	1.5499	ko04062<br>ko04742<br>ko04744	Chemokine signaling pathway<br>Photo transduction<br>Taste transduction
ATP synthase subunit O, mitochondrial	Atp5o	37.1	23.363	79	Up	0.623045921	3.108559973	1.6801	ko00190<br>ko05010	Alzheimer's disease<br>Huntington's disease

									ko05012<br>ko05016	Oxidative phosphorylation<br>Parkinson's disease
Lamin-B2	Lmnb2	31.7	67.317	126	Up	0.57357502	3.640287115	1.9653		
ATP synthase F(0) complex subunit B1, mitochondrial	Atp5f1	27.7	28.948	72	Up	0.409056664	3.447880438	1.8642	ko00190<br>ko05010<br>ko05012<br>ko05016	Alzheimer's disease<br>Huntington's disease<br>Oxidative phosphorylation<br>Parkinson's disease
Guanine nucleotide-binding protein subunit beta-4	Gnb4	2.9	37.379	36	Up	0.344211578	5.244639277	2.7145	ko04062	Chemokine signaling pathway
Guanine nucleotide-binding protein G(I)/G(S)/G(T) subunit beta-2	Gnb2<br>Gnb4	20.9	37.331	148	Up	0.33198595	2.675625039	1.4347	ko04062	Chemokine signaling pathway
Aconitate hydratase, mitochondrial	Aco2	24.6	85.462	123	Up	0.329710484	3.052902023	1.6492	ko00020<br>ko00630<br>ko00720	Carbon fixation pathways in prokaryotes<br>Citrate cycle (TCA cycle)<br>Glyoxylate and dicarboxylate metabolism
Non-POU domain-containing octamer-binding protein	Nono	37.8	54.54	133	Down	-0.165612221	-2.52636357	1.3477		
Tubulin beta-3 chain	Tubb3	13.3	50.418	256	Down	-0.256998539	-2.50070599	1.3326	ko04145<br>ko04540<br>ko05130	Gap junction<br>Pathogenic Escherichia coli infection<br>Phagosome
Vesicle-associated membrane protein 2; Vesicle-associated membrane protein 3	Vamp1<br>Vamp2<br>Vamp3	50.3	17.862	128	Down	-0.329986095	-2.971921974	1.6038	ko04130<br>ko04145<br>ko04962<br>ko04970	Phagosome<br>Salivary secretion<br>SNARE interactions in vesicular transport<br>Vasopressin-regulated water reabsorption
14-3-3 protein beta/alpha;14-3-3 protein beta/alpha, N-terminally processed	Ywhab	13.8	18.348	85	Down	-0.359491825	-2.755515905	1.4808	ko04110<br>ko04114<br>ko04722	Cell cycle<br>Neurotrophin signaling pathway<br>Oocyte meiosis
Serine/arginine-rich splicing factor 1	Srsf1<br>Srsf9	26.1	28.329	37	Down	-0.450505733	-2.726145759	1.4639	ko03040	Spliceosome
Heterogeneous nuclear ribonucleoproteins C1/C2	Hnrnpc	28.8	34.384	51	Down	-0.602185249	-3.918784687	2.1071	ko03040	Spliceosome
Haemoglobin subunit alpha	haemoglobin alpha 2; Hba	62	15.112	115	Down	-0.609102249	-2.938422415	1.5850	ko05143<br>ko05144	African trypanosomiasis<br>Malaria
14-3-3 protein gamma;14-3-3 protein gamma, N-terminally processed	Ywhag	30.8	28.302	160	Down	-0.657001495	-3.486308246	1.8846	ko04110<br>ko04114<br>ko04722	Cell cycle<br>Neurotrophin signaling pathway<br>Oocyte meiosis
Band 4.1-like protein3<br>Band 4.1-like protein 3, N-terminally processed	Epb4.1l3<br>Epb41l3	10.5	97.596	31	Down	-1.216077328	-2.96828511	1.6018	ko04530	Tight junction
Tropomyosin alpha-1 chain	Tpm1	11	28.343	35	Down	-1.237607479	-2.489495851	1.3261	ko04260	Cardiac muscle contraction

	Tpm2								ko05410<br>ko05414	Dilated cardiomyopathy<br>Hypertrophic cardiomyopathy (HCM)
Eukaryotic initiation factor 4A-II;<br>Eukaryotic initiation factor 4A-II, N-terminally processed;<br>Eukaryotic initiation factor 4A-I	Eif4a1<br>Eif4a2	6.6	46.402	28	Down	-1.270507813	-3.457459917	1.8693	ko03013	RNA transport
Ras-related C3 botulinum toxin substrate 1;<br>Ras-related C3 botulinum toxin substrate 3	Rac1<br>Rac2<br>Rac3	28	23.432	41	Down	-1.556331158	-2.702360444	1.4502	ko04010<br>ko04062<br>ko04145<br>ko04310<br>ko04360<br>ko04370<br>ko04380<br>ko04510<br>ko04520<br>ko04620<br>ko04650<br>ko04662<br>ko04664<br>ko04666<br>ko04670<br>ko04722<br>ko04810<br>ko04972<br>ko05014<br>ko05100<br>ko05120<br>ko05131<br>ko05200<br>ko05210<br>ko05211<br>ko05212<br>ko05416	Adherens junction<br>Amyotrophic lateral sclerosis (ALS)<br>Axon guidance<br>B cell receptor signaling pathway<br>Bacterial invasion of epithelial cells<br>Chemokine signaling pathway<br>Colorectal cancer<br>Epithelial cell signaling in Helicobacter pylori infection<br>Fc epsilon RI signaling pathway<br>Fc gamma R-mediated phagocytosis<br>Focal adhesion<br>Leukocyte trans endothelial migration<br>MAPK signaling pathway<br>Natural killer cell mediated cytotoxicity<br>Neurotrophin signaling pathway<br>Osteoclast differentiation<br>Pancreatic cancer<br>Pancreatic secretion<br>Pathways in cancer<br>Phagosome<br>Regulation of actin cytoskeleton<br>Renal cell carcinoma<br>Shigellosis<br>Toll-like receptor signaling pathway<br>VEGF signaling pathway<br>Viral myocarditis<br>Wnt signaling pathway
Glial fibrillary acidic protein	Gfap	24.4	49.899	36	Down	-1.846882343	-5.608623476	2.8632		
Heterogeneous nuclear ribonucleoprotein A0	Hnrnpa0	12.1	30.53	23	Down	-1.986347198	-4.562048267	2.4153		
Neurofilament medium polypeptide	Nefm	1.7	95.94	32	Down	-3.633944035	-3.699355388	1.9958	ko05014	Amyotrophic lateral sclerosis (ALS)
Rho-related BTB domain-containing protein 3	Rhobtb3	2.1	69.207	44	Down	-5.331884861	-3.050748316	1.6480		

Suppl. Table 5: The list of significantly differentially expressed protein, gene name, MS/MS count, unique sequence coverage, molecular weight (MW), T-test difference, p-value, various KEGG name, and pathways in Akita.PlGF$^{-/-}$ group compared to PlGF$^{-/-}$ group.

Protein names	Gene names	Unique sequence coverage [%]	Mol. weight [kDa]	MS/MS count	Regulation	Student's T-test Difference AkiPLGF$^{-/-}$_PLGF$^{-/-}$	Student's T-test Test statistic AkiPLGF$^{-/-}$_PLGF$^{-/-}$	-Log Student's T-test p-value	KEGG	KEGG name
Tubulin beta-6 chain	Tubb6	4	50.09	174	Up	0.511187553	2.707577783	1.4532	ko04145<br>ko04540<br>ko05130	Gap junction;<br>Pathogenic Escherichia coli infection;<br>Phagosome
Tubulin beta-3 chain	Tubb3	13.3	50.418	256	Up	0.422751904	2.53021607	1.3500	ko04145<br>ko04540<br>ko05130	Gap junction;<br>Pathogenic Escherichia coli infection;<br>Phagosome
Heat shock protein HSP 90-beta	Hsp90ab1	8.3	83.28	83	Up	0.342055798	2.509301016	1.3377	ko04141<br>ko04612<br>ko04621<br>ko04626<br>ko04914<br>ko05200<br>ko05215	Antigen processing and presentation;<br>NOD-like receptor signaling pathway;<br>Pathways in cancer;<br>Plant-pathogen interaction;<br>Progesterone-mediated oocyte maturation;<br>Prostate cancer;<br>Protein processing in endoplasmic reticulum
Tubulin beta-2B chain	Tubb2b	3.6	49.953	499	Up	0.264726639	2.471480938	1.3155	ko04145<br>ko04540<br>ko05130	Gap junction;<br>Pathogenic Escherichia coli infection;<br>Phagosome
Tubulin beta-5 chain	Tubb5	17.1	49.67	566	Up	0.258309364	2.611766276	1.3976	ko04145<br>ko04540<br>ko05130	Gap junction;<br>Pathogenic Escherichia coli infection;<br>Phagosome
Heat shock cognate 71 kDa protein	Hspa8	30.8	70.87	444	Up	0.156526566	2.95283795	1.5931	ko03040<br>ko04010<br>ko04141<br>ko04144<br>ko04612<br>ko05145<br>ko05162	Antigen processing and presentation;<br>Endocytosis;<br>MAPK signaling pathway;<br>Measles;<br>Protein processing in endoplasmic reticulum;<br>Spliceosome;<br>Toxoplasmosis
ATP synthase subunit O, mitochondrial	Atp5o	37.1	23.363	79	Down	-0.416332722	-2.562415443	1.3688	ko00190<br>ko05010<br>ko05012<br>ko05016	Alzheimer's disease;<br>Huntington's disease;<br>Oxidative phosphorylation;<br>Parkinson's disease
Microtubule-associated protein 1B;<br>MAP1B heavy chain;<br>MAP1 light chain LC1	Map1b	4.6	270.25	27	Down	-0.428781033	-2.496298553	1.3301		
MICOS complex subunit Mic60	Immt	10.2	83.899	62	Down	-0.451266289	-2.506606186	1.3361		
Heterogeneous nuclear ribonucleoprotein M	Hnrnpm	34	77.648	99	Down	-0.533585072	-3.107757295	1.6796	ko03040	Spliceosome
Voltage-dependent anion-selective channel protein 2	Vdac2	19.1	30.446	59	Down	-0.678982258	-2.85774511	1.5392	ko04020<br>ko05012	Calcium signaling pathway;<br>Huntington's disease;

									ko05016	Parkinson's disease
Transcriptional activator protein Pur-alpha	Pura	19.6	34.883	17	Down	-1.35616684	-3.491899783	1.8876		
T-complex protein 1 subunit theta	Cct8	10.8	53.082	21	Down	-1.527114391	-4.160793495	2.2262		
Cold-inducible RNA-binding protein	Cirbp	44.2	18.607	34	Down	-1.614155769	-2.464409392	1.3113		
Cytochrome c oxidase subunit 5B, mitochondrial	Cox5b	14.7	13.847	19	Down	-1.654263973	-3.035962803	1.6397	ko00190 ko04260 ko05010 ko05012 ko05016	Alzheimer's disease; Cardiac muscle contraction; Huntington's disease; Oxidative phosphorylation; Parkinson's disease
Methyl-CpG-binding protein 2	Mecp2	19	52.307	22	Down	-1.850307941	-3.542110193	1.9140		
Retinoschisin	Rs1	30.6	20.987	22	Down	-2.150679111	-3.433042051	1.8563		
NADH dehydrogenase [ubiquinone] 1 alpha subcomplex subunit 8	Ndufa8	37.2	19.992	26	Down	-2.18885231	-4.40456783	2.3423	ko00190 ko05010 ko05012 ko05016	Alzheimer's disease; Huntington's disease; Oxidative phosphorylation; Parkinson's disease
Enhancer of rudimentary homolog	Erh	46.5	8.2033	13	Down	-2.454516888	-2.978946933	1.6078		

Suppl. Figure 1: Quantitative profiling of retinal tissue between Akita.PlGF$^{-/-}$ and Akita groups.

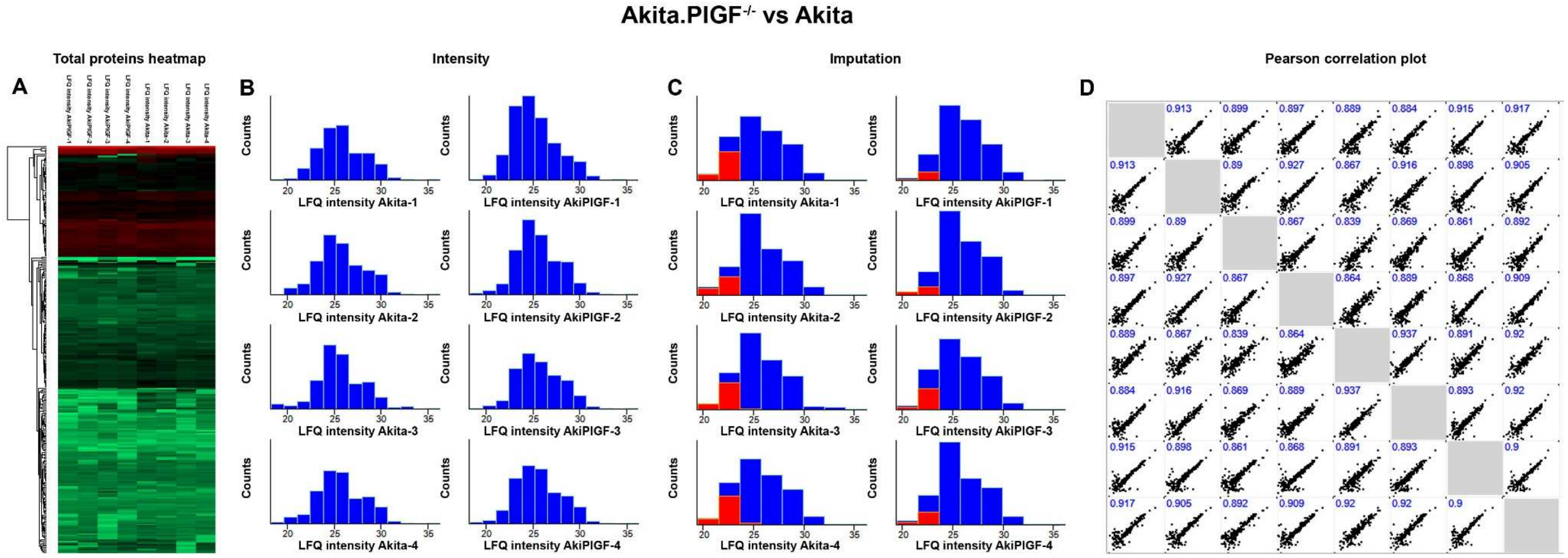

(**A**) Hierarchical clustering of Z-scored median LFQ intensities for all proteins in Akita.PlGF-/- vs. Akita groups. (**B**) LFQ intensity of each protein across Akita.PlGF$^{-/-}$ vs. Akita groups. (**C**) LFQ intensity of each protein across Akita.PlGF$^{-/-}$ vs. Akita group samples after imputation. (**D**) Pearson correlation coefficient of LFQ intensities among LC-MS/MS runs in Akita.PlGF$^{-/-}$ vs. Akita groups.

Suppl. Figure 2: Quantitative profiling of retinal tissue between Akita and C57 groups.

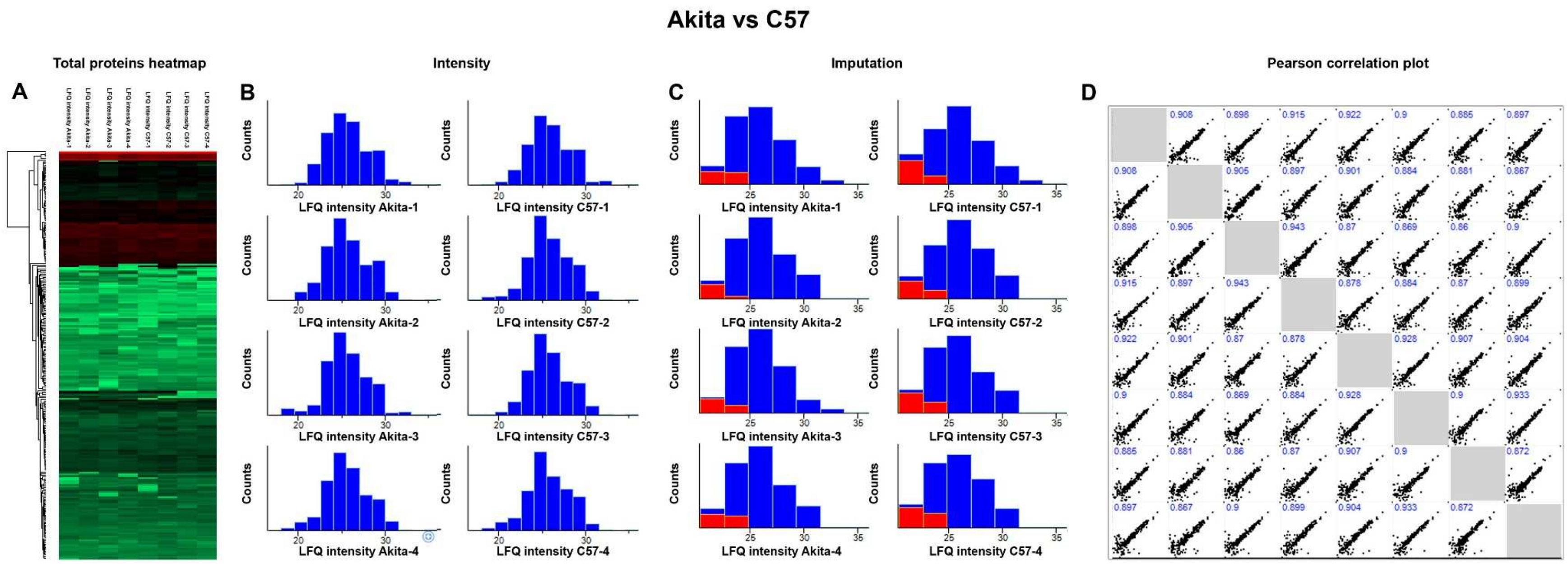

(**A**) Hierarchical clustering of Z-scored median LFQ intensities for all proteins in Akita vs. C57 groups. (**B**) LFQ intensity of each protein across Akita vs. C57 group samples. (**C**) LFQ intensity of each protein across Akita vs. C57 group samples after imputation. (**D**) Pearson correlation coefficient of LFQ intensities among LC-MS/MS runs in Akita vs. C57 groups.

Suppl. Figure 3: Quantitative profiling of retinal tissue between PlGF$^{-/-}$ and C57 groups.

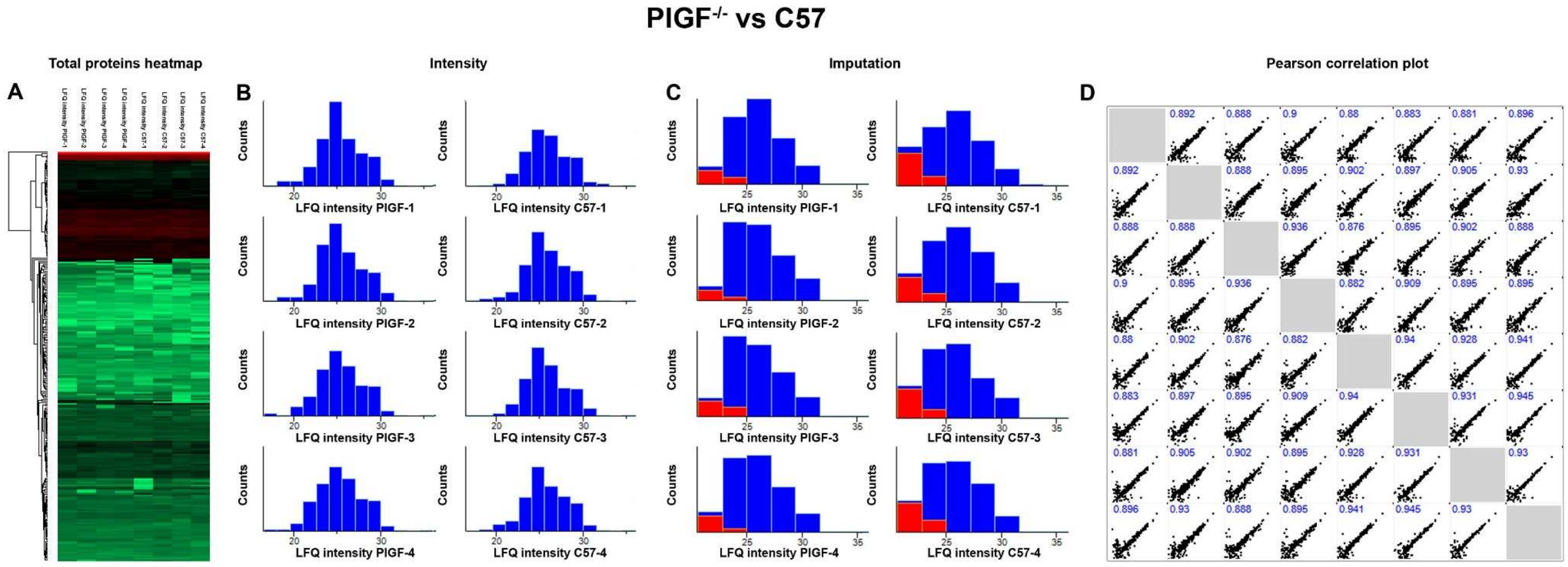

(**A**) Hierarchical clustering of Z-scored median LFQ intensities for all proteins in PlGF$^{-/-}$ vs. C57 groups. (**B**) LFQ intensity of each protein across PlGF$^{-/-}$ vs. C57 group samples. (**C**) LFQ intensity of each protein across PlGF$^{-/-}$ vs. C57 group samples after imputation. (**D**) Pearson correlation coefficient of LFQ intensities among LC-MS/MS runs in PlGF$^{-/-}$ vs. C57 groups.

Suppl. Figure 4: Quantitative profiling of retinal tissue between Akita.PlGF$^{-/-}$ and PlGF$^{-/-}$ groups.

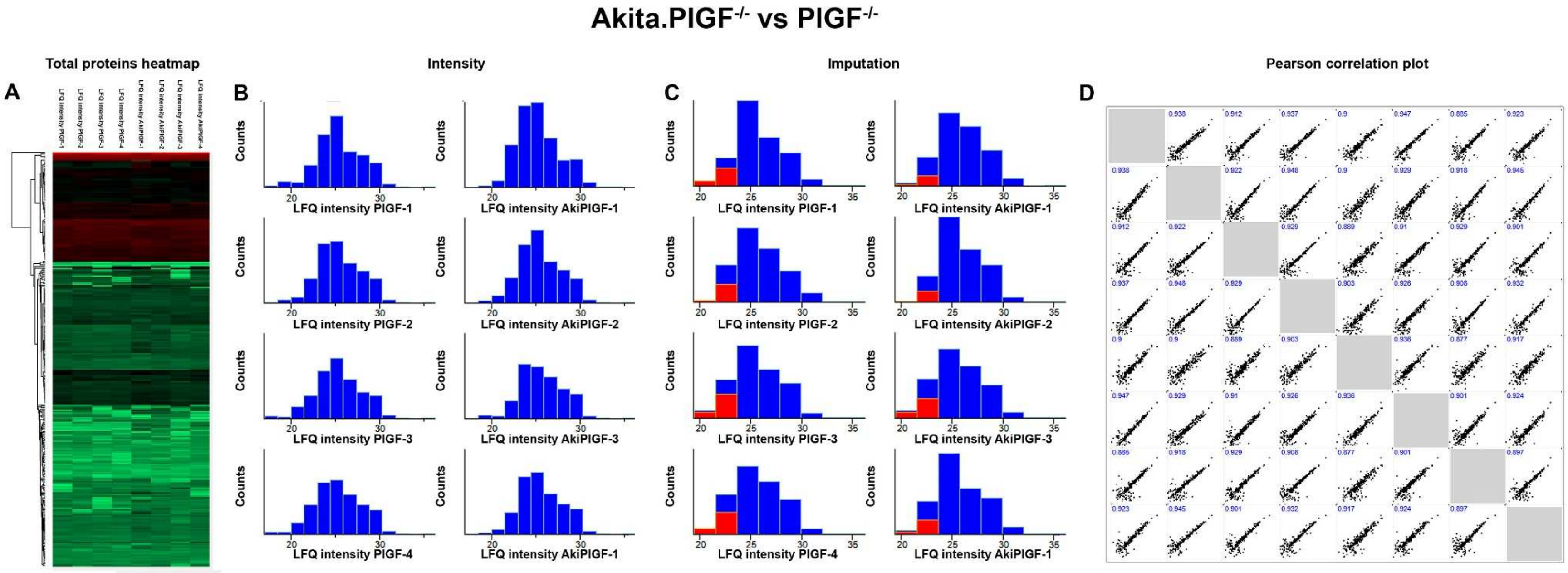

(**A**) Hierarchical clustering of Z-scored median LFQ intensities for all proteins in Akita.PlGF$^{-/-}$ vs PlGF$^{-/-}$ groups. (**B**) LFQ intensity of each protein across Akita.PlGF-/- vs. PlGF$^{-/-}$ group samples. (**C**) LFQ intensity of each protein across Akita.PlGF$^{-/-}$ vs. PlGF$^{-/-}$ group samples after imputation. (**D**) Pearson correlation coefficient of LFQ intensities among LC-MS/MS runs in Akita.PlGF$^{-/-}$ vs. PlGF$^{-/-}$ groups.

Suppl. Figure 5: Mass spectra of the representative insulin resistance, antioxidant and neural protection proteins.

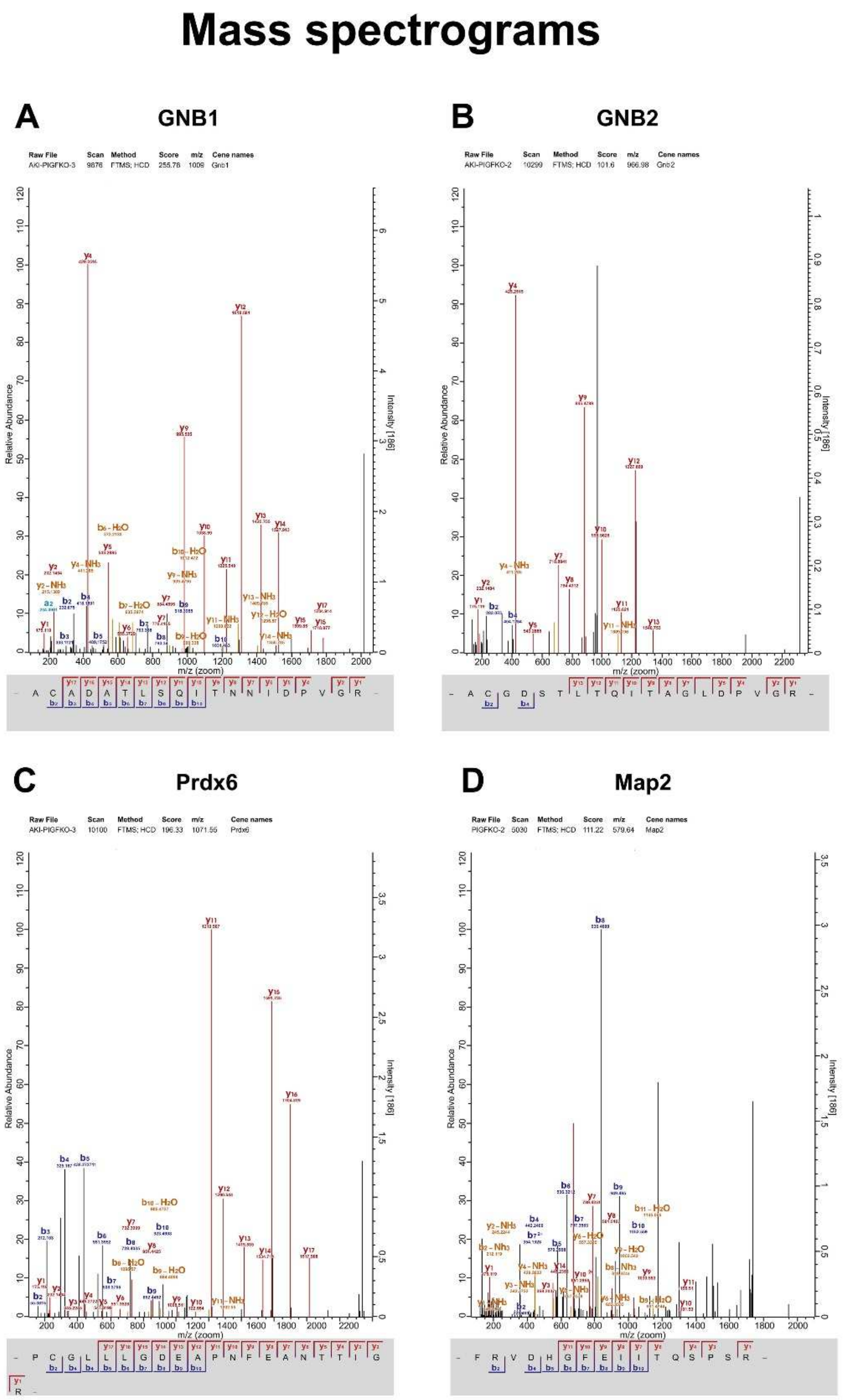

(**A)** Gnb1, (**B)** Gnb2, (**C)** Prdx6 and (**D)** Map2.
